# Thermal Performance of Hybrid-Inspired Coolant for Radiator Application

**DOI:** 10.3390/nano10061100

**Published:** 2020-06-02

**Authors:** F. Benedict, Amit Kumar, K. Kadirgama, Hussein A. Mohammed, D. Ramasamy, M. Samykano, R. Saidur

**Affiliations:** 1 Faculty of Engineering Technology Mechanical and Automotive, Universiti Malaysia Pahang, Pekan 26600, Malaysia; foojs1@gmail.com (F.B.); amithiamit@gmail.com (A.K.); kumaran@ump.edu.my (K.K.); deva@ump.edu.my (D.R.); 2 School of Engineering, Edith Cowan University, 270 Joondalup Drive, Joondalup, WA 6027, Australia; 3 Mechanical Department, College of Engineering, Universiti Malaysia Pahang, Pekan 26600, Malaysia; mahendran@ump.edu.my; 4 Research Center for Nano-Materials and Energy Technology (RCNMET), School of Science and Technology, Sunway University, Bandar Sunway, Petaling Jaya 47500, Selangor, Darul Ehsan, Malaysia; saidur@sunway.edu.my; 5 Department of Engineering, Lancaster University, Lancaster LA1 4YW, UK

**Keywords:** nanofluids, CNC, hybrid, thermal conductivity, heat transfer enhancement, car radiator

## Abstract

Due to the increasing demand in industrial application, nanofluids have attracted the considerable attention of researchers in recent decades. The addition of nanocellulose (CNC) with water (W) and ethylene glycol (EG) to a coolant for a radiator application exhibits beneficial properties to improve the efficiency of the radiator. The focus of the present work was to investigate the performance of mono or hybrid metal oxide such as Al_2_O_3_ and TiO_2_ with or without plant base-extracted CNC with varying concentrations as a better heat transfer nanofluid in comparison to distilled water as a radiator coolant. The CNC is dispersed in the base fluid of EG and W with a 60:40 ratio. The highest absorption peak was noticed at 0.9% volume concentration of TiO_2_, Al_2_O_3_, CNC, Al_2_O_3_/TiO_2_, and Al_2_O_3_/CNC nanofluids which indicates a better stability of the nanofluids’ suspension. Better thermal conductivity improvement was observed for the Al_2_O_3_ nanofluids in all mono nanofluids followed by the CNC and TiO_2_ nanofluids, respectively. The thermal conductivity of the Al_2_O_3_/CNC hybrid nanofluids with 0.9% volume concentration was found to be superior than that of the Al_2_O_3_/TiO_2_ hybrid nanofluids. Al_2_O_3_/CNC hybrid nanofluid dominates over other mono and hybrid nanofluids in terms of viscosity at all volume concentrations. CNC nanofluids (all volume concentrations) exhibited the highest specific heat capacity than other mono nanofluids. Additionally, in both hybrid nanofluids, Al_2_O_3_/CNC showed the lowest specific heat capacity. The optimized volume concentration from the statistical analytical tool was found to be 0.5%. The experimental results show that the heat transfer coefficient, convective heat transfer, Reynolds number and the Nusselt number have a proportional relationship with the volumetric flow rate. Hybrid nanofluids exhibit better thermal conductivity than mono nanofluids. For instance, a better thermal conductivity improvement was shown by the mono Al_2_O_3_ nanofluids than the CNC and TiO_2_ nanofluids. On the other hand, superior thermal conductivity was observed for the Al_2_O_3_/CNC hybrid nanofluids compared to the other mono and hybrid ones (Al_2_O_3_/TiO_2_).

## 1. Introduction

Heat transfer has been an important criterion for many industrial types of equipment and machinery. Many industrial facilities and machineries require proper heat transfer management from different phases for efficient productivity [1,2]. Thus, an efficient heat transfer fluid is required for optimal heat management and efficient process. Correspondingly, the swift increase in energy demand further necessitates the need for enhancement in the heat transfer process and reduction in energy loss due to the inept use of the system. Specifically, in an automotive application, coolants and oils are used as heat transfer mediums. As the name implies, a coolant plays a significant role in reducing the heat in the automotive components. Currently, several different radiator liquid coolant fluids are available in the market which differ from each other in terms of heat assimilation limit, capacity and heat transfer properties. For example, water is one of the common fluids which has great heat-exchanging qualities and is abundantly available. It is classified as a perfect coolant considering its capacity to ingest and discharge heat efficiently [3]. Furthermore, water is very viscous, which allows it to flow easily and quickly in the system enabling it to be utilized as a radiator coolant [4]. Ethylene glycol (EG) is also used extensively as an automotive antifreeze during both summer and winter seasons due to its high boiling points [5]. Diethylene glycol (DEG) and propylene glycol (PEG) are also commonly utilized as antifreeze in automobiles. These are miscible in water, alcohol, ether, acetone and ethylene glycol, and thus are widely used as solvents [6]. The main function of these liquids is to absorb and dispose of the heat generated by the car engine. However, the aforementioned coolants have inadequate heat transfer properties, thus decreasing the performances of the automobile components and its efficiency. Basically, a coolant with a high heat transfer limit, a minimal effort to the water pump, low viscous fluid and that is synthetically inert usually desired and considered as a perfect coolant. As such, it has paved the development of a new fluid called nanofluids which are expected to have that high thermal performance. 

Nanofluids can be defined as the dispersion of nanometer-sized solid metal or metal oxide particles in single phase fluids. The thermo-physical properties of nanofluids were found to be superior compared to the single-phase coolant in the aforementioned applications, since nanoparticles exhibit a higher thermal conductivity in comparison to the base fluids and result in the significant enhancement of the thermal conductivity and heat transfer performance of the base fluids. Several metal or metal oxide nanoparticles such as Al_2_O_3_, Ag, CuO, Cu, Co_3_O_4_, Fe_3_O_4_, Fe_2_O_3_, SiC, SiO_2_, TiO_2_, ZnO, nano-diamond, graphite and carbon-nanotubes (CNT) have been used and reported for the preparation of nanofluids. Furthermore, as per the literature, the thermo-physical properties such as the viscosity, density and the thermal conductivity of nanofluids also depends on the temperature, size and concentration of nanoparticles [7]. For example, Dhaiban [8] studied the thermal properties of zinc dioxide–ethylene glycol (ZnO_2_–EG)-based nanofluids and observed a 26.5% improvement in thermal conductivity by adding only a 5% volume fraction of ZnO_2_ nanoparticles into the base fluid (EG). An investigation on convective heat transfer measurement using Al₂O₃/water nanofluids also revealed the improvement in the heat transfer coefficient as the concentrations of nanoparticles increased [9]. Mintsa, Roy [10] observed that nanofluids containing 170 nm silicon carbide nanoparticles (3.7% volume concentration) showed an improvement of up to 50%–60% in the Reynolds number and the heat transfer coefficients. Another study using copper oxide/water-based nanofluids also revealed an improvement in the heat transfer coefficient when the volume concentration used was between 0–4% [11]. The thermal performance of a coil heat exchanger having 97.5% alumina and 2.5% Ag prepared at 0.1–0.4% concentration was investigated by Allahyar, Hormozi [12] and the maximum heat transfer was reported for the 0.4% concentration of the hybrid/composite nanofluids. 

As per the above literature discussion, it can be concluded that a small dispersion of a single content nanoparticle in a base fluid can enhance the thermal conductivity of the nanofluid. It is also evident to expect that the dispersion of two or more content nanoparticles in a base fluid can exhibit a better performance than the conventional fluid as well as the nanofluid with a single nanoparticle. In view of this, some research groups have reported that hybrid/composite nanofluids exhibit a better performance than the single-phase system. The term hybrid/composite is widely used in the field of nanotechnology. Basically, these are the composites which consist of at least two components at the nanometer or molecular level. Hybrid/composite nanofluids have a better stability as well as a superior performance. Their thermal conductivity can be increased by dispersing even a small quantity of nanoparticles in the base fluid and considered as a promising heat transfer fluid for the future. Sidik, Yazid [13], Li, Zou [14] and Philip, Shima [15] reported that nanofluids are advanced kind of fluids which contain a small quantity of nanoparticles—particles which have a diameter less than 1–100 nm and uniformly suspended in a liquid. Recently, a few research articles have been published on hybrid/composite nanofluids containing mixed ratios of nanoparticles [16]. For example, Afrand, Toghraie [16] prepared the Fe_3_O_4_–Ag (1:1 ratio) water-based hybrid/composite nanofluid with different solid volume fractions and investigated the effect of temperature and the concentration of nanoparticles on the rheological behavior. They evaluated the viscosity in the temperature range of 25–50 °C and observed that the viscosity of the Fe_3_O_4_–Ag water-based hybrid/composite nanofluid decreased with the increment of the fluid temperature. Furthermore, the hybrid/composite nanofluid containing less than a 0.3% solid volume fraction showed Newtonian behavior, however, others exhibited non-Newtonian behavior. Similar studies have also been reported by Bahrami, Akbari [17] for Fe and CuO with an equal ratio (1:1) in water/ethylene glycol-based hybrid/composite nanofluids at different solid volumes. Ahammed, Asirvatham [18] prepared 0.1% graphene–alumina with a 1:1 ratio hybrid/composite nanofluids in water as a base fluid and investigated their entropy generation as did other authors [19,20,21]. They have also compared the performance of a graphene-alumina hybrid/composite with alumina water-based single nanofluids. Esfe, Afrand [22] also prepared Ag–MgO (1:1 ratio) water hybrid nanofluids at concentrations of 0.0%, 0.005%, 0.01%, 0.015% and 0.02%, and observed that all the hybrid nanofluids remained stable for several days. On the other hand, Kumar, Vasu [23] and Huang, Wu [24] prepared Cu–Zn (1:1) hybrid nanofluids and Al_2_O_3_–MWCNT (1:2.5) hybrid nanofluids which were stable for two weeks. 

Along with the advanced properties of metal oxides for nanofluid preparation, crystalline nanocellulose (CNC) has also received significant attention for its potential application in the automotive field or in nanofluid preparation. CNC is a nature-based nanomaterial (natural material) that has a superior material behavior, especially at the nanoscale to be used in various applications. CNC has also been reported to have a great advantage in terms of sustainability, abundance, mechanical properties such as a large surface to volume ratio, high-tensile strength and stiffness, high flexibility as well as good electrical and thermal properties. Moreover, CNC is also a safe and environmentally friendly material to handle. Therefore, the focus of the present work was to investigate the performance of a mono or hybrid metal oxide such as Al_2_O_3_ and TiO_2_ with or without plant base-extracted CNC in varying concentrations as a better heat transfer nanofluid in comparison with readily available coolants, namely EG-distilled water as a radiator coolant. The results of different nanofluids on the car radiator performance are discussed and interpreted in this paper. 

## 2. Preparation of Nanofluids

The parameters such as the concentration volume, the required volume of nanofluid and the amount of cellulose to be mixed with the nanofluid were calculated and finalized before the material preparation. The fluid was prepared by mixing water and EG at a certain percentage. The volume of the prepared mixture was equal to the total volume of the radiator during the standard procedure in an automotive engine. In the present study, the radiator with the volume of 4 L was used for observation. Accordingly, 3.9 L of the nanofluid was prepared for 0.5% volume concentration. The volumes of the nanocellulose and the metal oxide were determined by using the following Equations (1) and (2), [13,14,15]:

(1) $$\emptyset =\frac{\omega \rho_{w}}{\left(1-\frac{\omega }{100}\right)\rho_{p}+\frac{\omega }{100}\rho_{w}}$$

(2) $$\Delta V=\left(V_{2}-V_{1}\right)=V_{1}\;\left(\frac{\emptyset_{1}}{\emptyset_{2}}-1\right)$$

Before the synthesis, all the glassware such as the beaker, the measuring cylinder and the syringe were cleaned properly. In a typical procedure, firstly, 368 mL of distilled water was taken in a 1 L beaker followed by 552 mL of the EG. The ratio of water to EG was 40:60. Furthermore, 70 mL nanocellulose was dispersed in the above base fluid of distilled water and EG using a syringe. The mixture was magnetically stirred for 30 min to ensure the homogeneous dispersion of the nanocellulose in water and the EG solution. Finally, the mixture was sonicated for 2 h at 50 °C temperature.

### 2.1. Material Characterization

**Figure 1:**
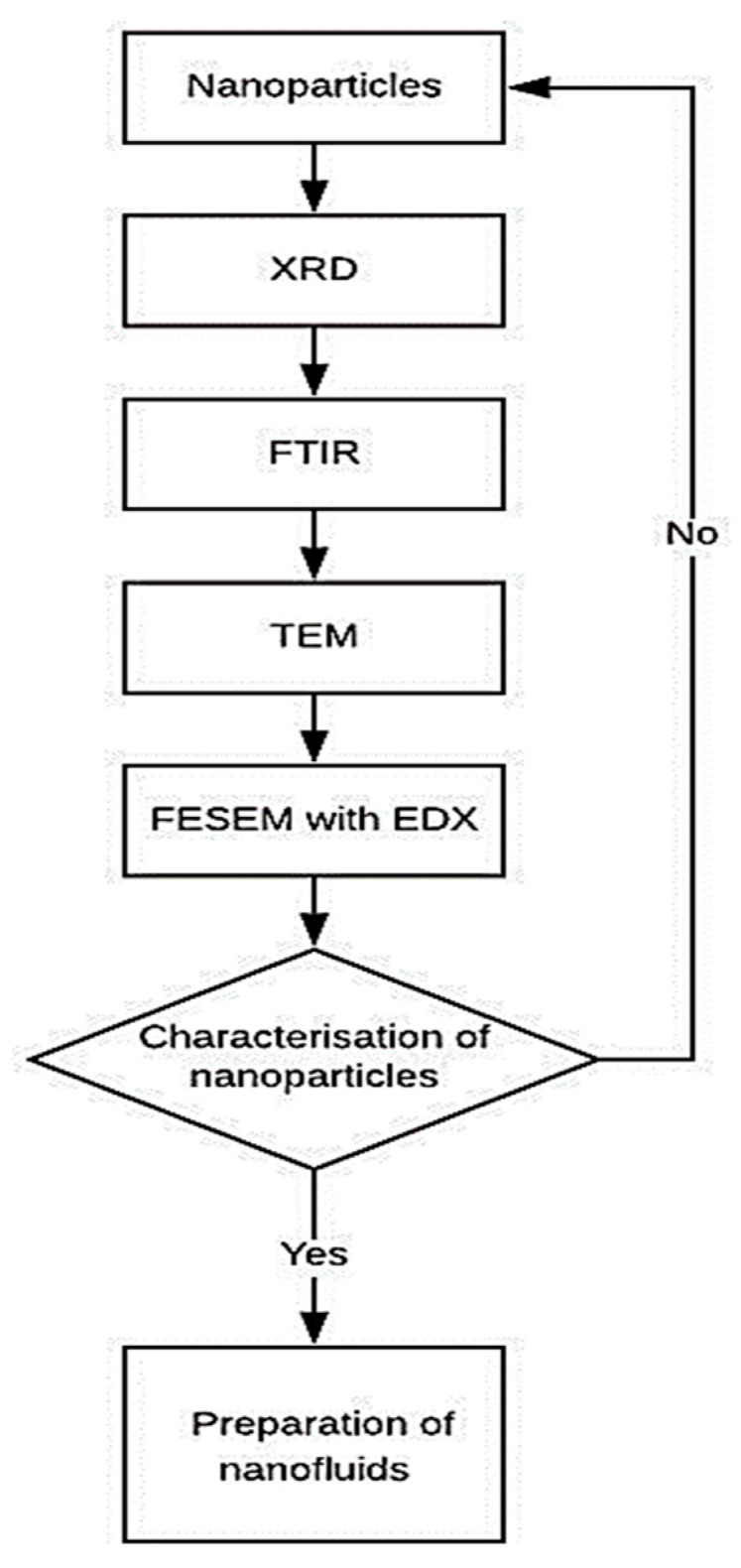
Flow chart of the material characterization, FTIR = Fourier Transform Infrared Spectroscopy; EDX=Energy-dispersive X-ray spectroscopy.

The phase and crystal analysis of the prepared compounds and composites were examined by recording the X-ray diffraction (XRD) patterns using an X-ray diffractometer. Fourier-transform infrared (FTIR) spectra were recorded to identify the chemical composition such as the functional groups present in the samples as prepared compounds and composites using the FTIR spectrometer. The spectra were obtained by making pallets of all the aforementioned compounds with Potassium Bromide, KBr. The morphological examination was carried out by capturing the topographical images of the compounds and the composites using Field Emission Scanning Electron Microscope (FESEM) and Transmission Electron Microscope (TEM). For the TEM analysis, the sample was dispersed in distilled water using an ultrasonicator for 15 min and then the suspension was poured on to a carbon-coated copper grid (200 meshes) and air dried. Energy-Dispersive X-ray spectroscopy (EDX) was also done during the FESEM measurement to investigate the elemental analysis present in the samples. The characterization of the materials’ procedure is shown in Figure 1.

### 2.2. Thermo-Physical Characterization

The thermal properties such as the density, specific heat capacity, thermal conductivity and the viscosity of the nanofluids depend upon various processes and system parameters such as preparation method and surface chemistry of the nanoparticles. Furthermore, the stability of the nanofluids, which is a very important factor, also depends on the preparation method.

Density (ρ_nf_) is a measure of how heavy an object is for a given size, i.e., the mass of material per unit volume and it does not depend on the amount or shape of the material, but it varies with the temperature and pressure. Firstly, the base nanofluids have been identified by measuring the densities and comparing its results with the standard values [19]. The density of nanofluid was calculated by using Equation (3) (Wang, Xu [25], Wen and Ding [26], Heris, Esfahany [9], Trisaksri and Wongwises [27], Zhou and Ni [28],Williams, Buongiorno [29], Das, Narayan [30], Duangthongsuk and Wongwises [31], Demir, Dalkilic [32], Sharma, Sarma [33],Fedele, Colla [34], Kayhanian, McKenzie [35], Garnett [36], Mahmud, Das [20], Tasnim, Mahmud [21], Das, Li [37]):

(3) $$\rho_{\mathrm{nf}}=\emptyset \rho_{p}+\left(1-\emptyset \right)\rho_{f}$$

The performance of the base fluid (water, EG, 10% EG–90% water and 20% EG–80% water) has also been compared with the standard values given by (ASHRAE, 2005). 

The specific heat capacity (*C*_nf_) (which is the total amount of heat required to increase the temperature of a system) of nanofluids was estimated by using Equation (4): (Lee, Choi [38], Duangthongsuk and Wongwises [31], Putra, Roetzel [39], Trisaksri and Wongwises [27], Wen and Ding [26], Heris, Esfahany [9], Zhou and Ni [28],Williams, Buongiorno [29], Sharma, Sarma [33], Fedele, Colla [34], Gosselin and da Silva [40], Chang, Su [41], Garnett [36], Mahmud, Das [20], Tasnim, Mahmud [21], Das, Li [37]).

(4) $$C_{\mathrm{nf}}=\emptyset C_{p}+\left(1-\emptyset \right)C_{f}$$

where *C*_nf_, *C*_f_ and *C*_p_ are the specific heat of the nanofluid, the base fluid and the nanoparticles, respectively. Initially, the device was calibrated by measuring the specific heat capacity of the standard fluid (glycerin) and then the measurements were carried out on the nanofluid and base fluid.

The transient hot-wires process was used to measure the thermal conductivity of the nanofluids. The wire was to be used as a line heat source, so the wire diameter was usually kept within 100 µm. The length of the wire was kept to just a few centimeters, which compared to the wire diameter represents an infinitely long line of heat source, assuring one directional (radial) heat transfer. The calibration process was also done with the standard fluid (glycerin) before the measurement. In the present work, three models were adopted to determine the thermal conductivity of the nanofluid. The first one was the Maxwell model used for the solid–liquid mixture with relatively large particles. It is based on the solution of the heat conduction equation through a stationary random suspension of spheres [42] as per Equation (5):

(5) $$\frac{k_{\mathrm{nf}}}{k_{f}}=\frac{k_{p}+2k_{f}+2\emptyset \left(k_{p}-k_{f}\right)}{k_{p}+2k_{f}-\emptyset \left(k_{p}-k_{f}\right)}$$

The second one is Bruggeman’s model to study the interactions between randomly distributed spherical particles [43] as

(6) $$\frac{k_{\mathrm{nf}}}{k_{f}}={\left[\left(3\emptyset -1\right)\frac{k_{p}}{k_{f}}+\left(3\left(1-\emptyset \right)-1\right)\right]}^{2}+8\frac{k_{p}}{k_{f}}$$

The last model is the Hamilton–Crosser model which is used for non-spherical particles. The model is based on the thermal conductivity of both the base fluid and the particle, the volume fraction and shape of the particles [44] as per Equation (5):

(7) $$\frac{k_{\mathrm{nf}}}{k_{f}}=\frac{k_{p}+\left(n-1\right)k_{f}+\left(n-1\right)\emptyset \left(k_{p}-k_{f}\right)}{k_{p}+\left(n-1\right)k_{f}-\emptyset \left(k_{p}-k_{f}\right)}$$

Viscosity is another important factor to evaluate the thermal properties of nanofluids. A commercial Brookfield DV-I prime viscometer (Brookfield DV-I USA) was used to measure the viscosity of the nanofluids at different temperatures and rotor speeds (rpm). This type of viscometer is generally used for Newtonian and non-Newtonian liquids having low- to high-viscosity values (depending on the spindle, from 1 to 600 cP). 

## 3. Experimental Section

### 3.1. Test Rig Setup

**Figure 2:**
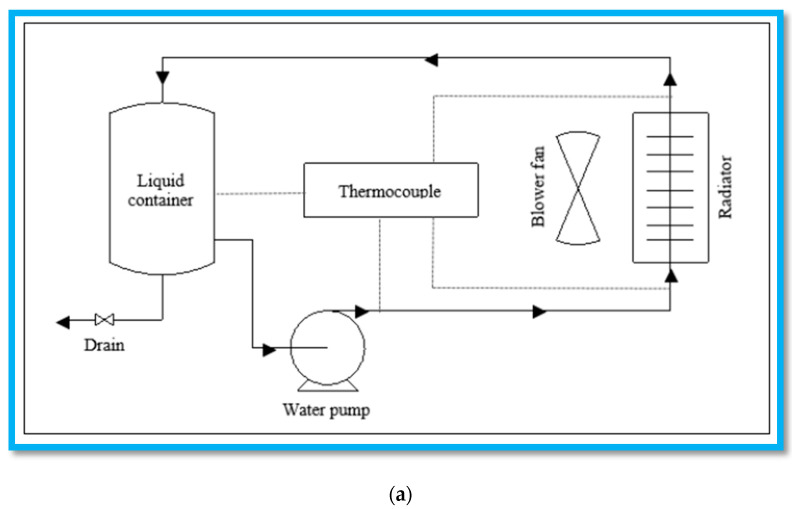

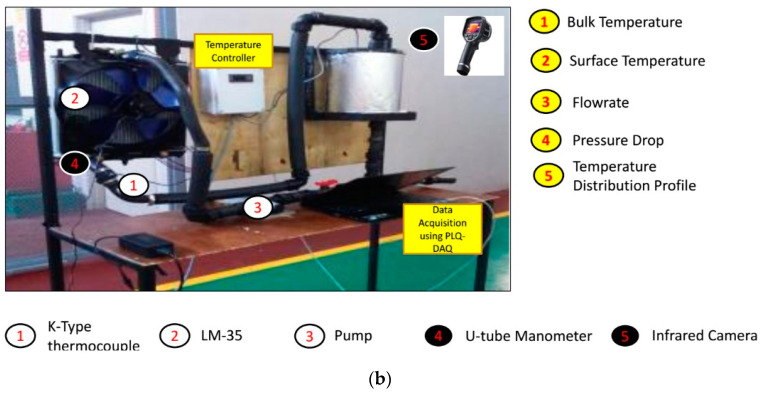
(**a**) Schematic diagram of the radiator test rig, (**b**) Experimental set-up of the radiator test rig. Parallax Data Acquisition tool (PLQ-DAQ); Integrated analog temperature sensor whose electrical output is proportional to Degree Centigrade (LM-35).

The testing of all the prepared nanofluids was carried out using a radiator test rig setup. The schematic diagram of the used radiator test ring is shown in Figure 2a,b. A 24 V DC supply was used as the main power source for the pump and heater. The radiator test rig was a closed loop system where the water circulated in the system by water pump. There were K-Type thermocouples (1.5 mm, USA) at four points on the radiator wall to measure the surface temperature of the radiator. A 12 V cooling fan (I-COOL, Japan) was attached to the radiator which acts as a normal radiator fan like a readily available automobile radiator. The heat produced by an automobile system during its routine was imitated by a 1kW heater (Dernord, USA). The volume of the essential coolant fluids was 4 L, composed of nanofluid and distilled water and stored in a 5 L metal tank. 

There are some parameters which are kept constant and manipulated during the radiator test rig experiments. Table 1 reports the variables that are responsible in determining the result obtained during the radiator test rig experiments.

Other parameters such as the Reynolds numbers, the Prandtl number and the Nusselt number which are generally used in fluid mechanics to characterize the heat transfer and fluid flow behavior were also discussed in the present work. 

## 4. Results and Discussion 

### 4.1. Physical, Chemical and Morphological Characterization

Generally, the agglomeration and rapid settling of particles are some of the problems faced by suspended particles in the fluid [45]. Although the heat transfer enhancement directly depends upon the high durability and the better stability of suspended particles in the fluid, in the present work, the sonication process was used for the preparation and control of the stability of the nanofluids. Duangthongsuk and Wongwises [31] prepared more stable nanoparticles without any agglomeration by increasing the time of the sonication process. They observed that the test solutions containing a fixed volume ratio of the base fluid (EG: W) with different volume concentrations were highly stable for more than one month. The sedimentation observation of all the samples i.e., Al_2_O_3_, TiO_2_ nanoparticle, nanocellulose CNC, the hybrid (Al_2_O_3_ + TiO_2_) nanoparticle and hybrid (Al_2_O_3_ + CNC) nanocomposite after six weeks are shown in Table 2. 

Supernatant concentration is also an important factor to control the stability of a nanofluid. In the present work, Al_2_O_3_/CNC and CNC were prepared without using any surfactant and we found that the solutions remained stable with minimum sedimentation even after one month. The nanofluids were also found to be stable during the thermo-physical investigation and the force convection experiment. Similar results were also found by Rao, Sreeramulu [46] who reported that nanofluids can remain stable for up to three months by increasing the timing of the ultra-sonication process. Ra, Sreeramulu [46] and Maheshwary and Nemade [47] reported thorough investigations on the effect of the sonication process for the synthesis of ZrO_2_/water nanofluids. They obtained some surprising results where the sonication process-routed nanofluid exhibited a better thermal conductivity enhancement and it was suitable for cooling applications. Furthermore, the observation for more than one month indicated that the nanofluid displayed a small amount of sedimentation in all base fluids which may be due to the gravitational forces. The stability of the Fe_3_O_4_ nanoparticles dispersed in a water–ethylene glycol mixture lasting up to one month was also reported by Sundar, Singh [48]. Upon aging, the particle aggregates may be due to high surface activity, as reported by Mohamed, Sagisaka [49]. In the present work, it was observed that the sedimentation occurred in the samples after six weeks.

**Figure 3:**
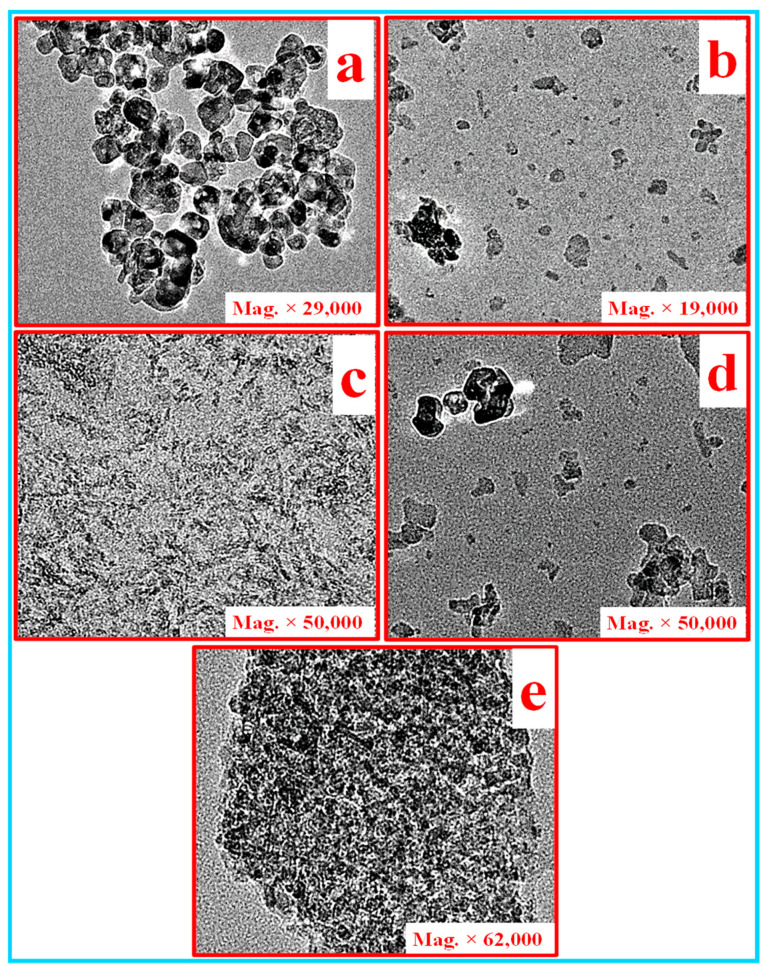
TEM images of, (**a**) TiO_2_, (**b**) Al_2_O_3_, (**c**) CNC, (**d**) Al_2_O_3_/TiO_2_ and (**e**) Al_2_O_3_/CNC nanofluids.

A transmission electron microscope (TEM) was used to acquire the high-resolution images of Al_2_O_3_, CNC, TiO_2_, TiO_2_ + Al_2_O_3_ and Al_2_O_3_ + CNC in the nanofluid with a high magnification and the results are shown in Figure 3a–e, respectively. However, the contrast and resolution were limited while acquiring the image of Al_2_O_3_ and CNC which may be due to low electron densities and a low profile [50,51]. Figure 3a shows the TEM image of the dispersed TiO_2_ nanoparticles into the ethylene glycol–water mixture (EG–W) fluid which illustrates that primary TiO_2_ particles have an almost uniform morphology and are interconnected to each other. However, the particles seemed to be nearly homogeneously dispersed in the base fluid. The TEM image of the Al_2_O_3_ nanoparticles dispersed evenly into the base fluid is displayed in Figure 3b which illustrates that the particles are almost uniformly dispersed in the base fluid with very small aggregation. The TEM micrograph of the CNC nanoparticles dispersed in base fluid is represented in Figure 3c. It can be clearly seen that the CNC nanoparticles completely homogeneously dispersed in the fluid, which is the one of the main requirements of the present application. Figure 3d,e shows the TEM micrographs of the Al_2_O_3_/TiO_2_ and Al_2_O_3_/CNC hybrid nanofluids, respectively. It can be observed that the dispersion of the Al_2_O_3_ and TiO_2_ nanoparticle is approximately uniform in the base fluid, however, both types of nanoparticle were not completely interconnected to each other. On the contrary, it can be clearly seen in Figure 3e that the Al_2_O_3/_CNC hybrid nanofluids were dispersed uniformly in the fluid. Furthermore, the Al_2_O_3_ and CNC nanoparticles were completely interconnected to each other i.e., the agglomerated particles which resulted in strong stability enhancement. Philip, Shima [15] reported that the formation of the agglomerated particles in the nanofluid basically depends upon on the surface contact between the particles. A strong van der Waals force works between the agglomerated particles which is very hard to break it into primary nanoparticles.

**Figure 4:**
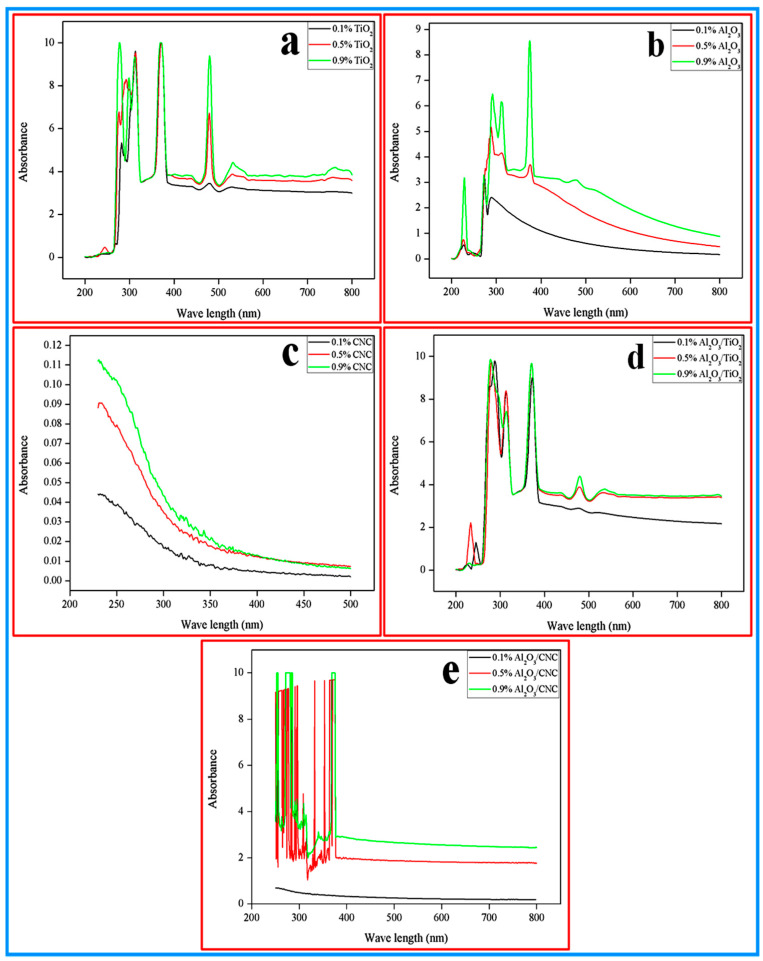
UV spectrum of (**a**) TiO_2_, (**b**) Al_2_O_3_, (**c**) CNC, (**d**) Al_2_O_3_/TiO_2_ and (**e**) Al_2_O_3_/CNC nanofluids with various concentrations.

Ultraviolet–visible spectrophotometer (UV–Vis) was used to evaluate the stability of the nanoparticles dispersed in the base fluids. The UV–Vis spectrum of all the prepared nanofluids with all the volume concentrations were recorded in the wavelength range of 200–800 nm and the results are shown in Figure 4a–e. It can be observed from all the UV–Vis spectra that among all the concentrations of all nanofluids, i.e., the TiO_2_, Al_2_O_3_, CNC, Al_2_O_3_/TiO_2_ and the Al_2_O_3_/CNC nanofluids, 0.9% concentration exhibited the maximum absorption peak, indicating the better stability of the nanofluid suspension. It was also noticed that the maximum absorption peak appeared in range of a 200–400 nm wavelength for all the nanofluids with all the volume concentrations. However, the range was found to be in a 200–250 nm wavelength in the case of the CNC nanofluids with all the volume concentrations. Furthermore, there was no absorption peak noticed for 0.1% Al_2_O_3_/CNC nanofluids which may be due to the instability of the nanofluid dispersion. Richardson and Zaki [52] also observed and reported similar behavior in nanofluids which may be because of the adjacent particle. After the formation of a colloidal suspension, the base fluid creates an upward stream which pushes the nanoparticles and prevents them from falling due to the gravity acceleration. Hence, the upward stream impact is greater in a high concentration than a low concentration nanofluid which reduces the absorbance drop in the colloidal suspension.

**Figure 5:**
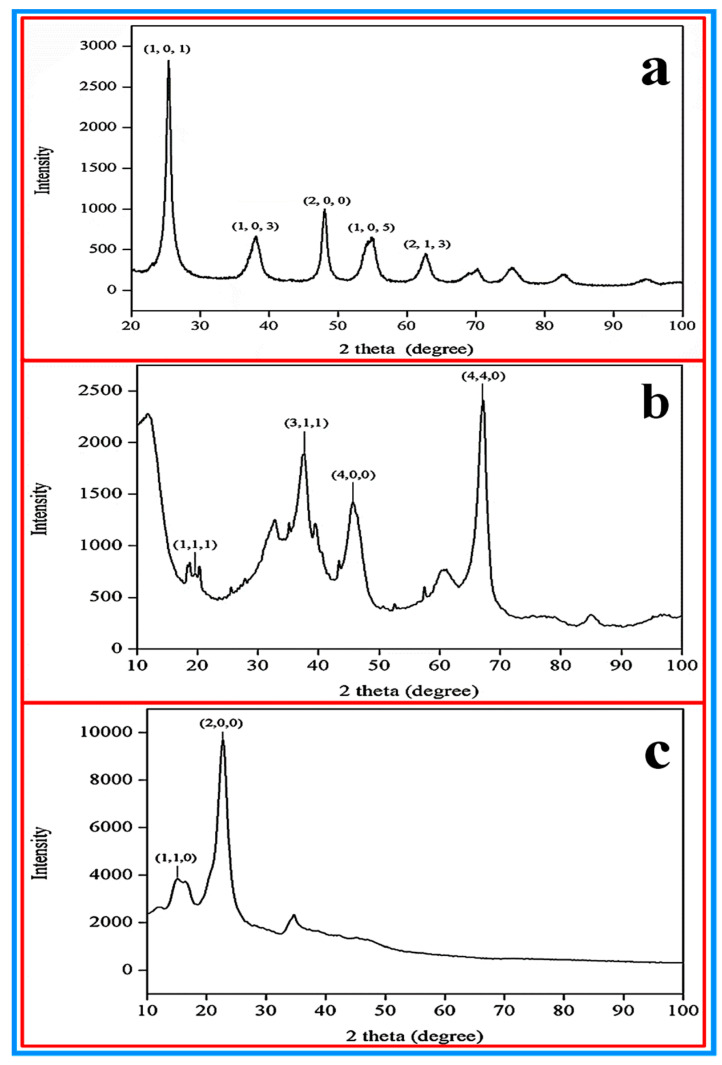
XRD patterns of (**a**) TiO_2_ and (**b**) Al_2_O_3_ nanoparticles, and (**c**) CNC.

**Figure 6:**
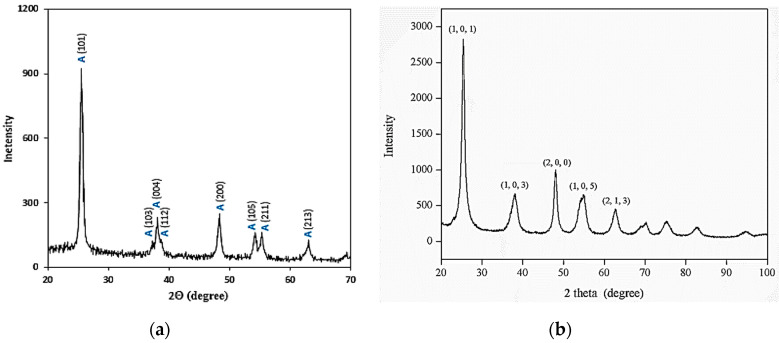
Comparison of the XRD patterns of the TiO_2_ nanoparticles from (**a**) Al-Taweel and Saud et al. [53] and (**b**) the current study. Figure 6a is reproduced with permission from [Al-Taweel, S.S.; Saud, H.R. New route for synthesis of pure anatase TiO_2_ nanoparticles via ultrasound assisted sol-gel method, Published by (*J. Chem. Pharm. Res.*) 2016].

**Figure 7:**
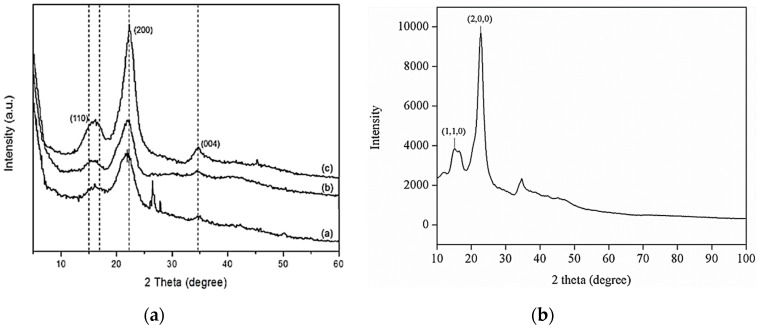
Comparison of the XRD patterns of the CNC nanoparticles from (**a**) Kumar, Negi [55] and (**b**) the current study. Figure 7a is reproduced with permission from [Kumar, A.; Negi, Y.S.; Choudhary, V.; Bhardwaj, N.K. Characterization of cellulose nanocrystals produced by acid-hydrolysis from sugarcane bagasse as agro-waste, Pubslihed by Journal of Materials Physics and Chemistry, 2014].

The crystal structure information of all the samples was collected by recording and analyzing the obtained XRD patterns. The XRD patterns of the TiO_2_, Al_2_O_3_ and the CNC nanoparticles are shown in Figure 4a–c, respectively. The XRD pattern of TiO_2_ is displayed in Figure 5a where all the characteristic peaks i.e., at 2θ angles of 25.28°, 37.93°, 48.37°, 53.88° and 62.72° correspond to the (101), (103), (200), (105) and (213) respectively, are in good agreement with the standard XRD pattern (ICDD no. 00-001-0562) and consistent with what was reported by Al-Taweel and Saud [53], which is portrayed in Figure 6. Figure 4b shows that the alumina phase which was identified at 2θ values of 19.4°, 37.7°, 45.8° and 66.8° which correspond to the diffraction from the (111), (311), (400) and (440) crystal planes, respectively; these results agreed with the standard XRD pattern (ICDD, PDF no. 01-074-2206 (Al_2_O_3_) 5.3333 Aluminum Oxide) according to [54]. The XRD pattern shown in Figure 4c reveals the pure phase of the CNC (C_6_H_10_O_5_)*_n_* Cellulose-1ß)) nanoparticles where most intense peaks at 2θ angles of 16.6° and 22.9° correspond to the (1, 1, 0) and (2, 0, 0) crystal planes, respectively, and other peaks are well-matched with the standard XRD pattern (ICDD no. 00-056-1718) and in accordance with Kumar, Negi [55], which is shown in Figure 7. 

**Figure 8:**
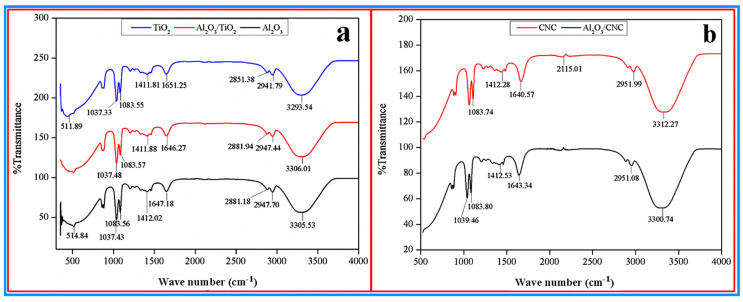
FTIR spectra of **(a)** TiO_2_, Al_2_O_3_, Al_2_O_3_/TiO_2_ (hybrid) nanofluids, and (**b**) CNC mono and Al_2_O_3_/CNC hybrid nanofluid.

The FTIR spectra were recorded to investigate the chemical composition of the mono and hybrid nanofluids and the results are shown in Figure 8a,b, respectively. It can be noticed from both figures that the FTIR spectra for all the mono and hybrid nanofluids were almost identical. All the spectra of the nanofluids contain a broadband in the frequency range of 3200 to 3650 cm^−1^ and one sharp band at 1640 cm^−1^ which can be attributed to the stretching and bending mode of the O-H group of EG and water, respectively. The band at around the 2950 cm^−1^ wave number in all the spectra may correspond to the stretching of the C–H groups of EG [53,56,57]. The band found at 1412 cm^−1^ may correspond to the CH_2_ stretching of EG. On the other hand, the band at 2115 cm^−1^ can be noticed in the spectra of the CNC and Al_2_O_3_/CNC nanofluids, which can be ascribed to the C≡C bonds. From both Figure 8a,b, it can be observed that no band was noticed for the metal oxide (Al_2_O_3_ and TiO_2_) in all the spectra. Besides that, all the bands corresponded to only the EG with water and the CNC chemical composition. Therefore, it can be concluded that no chemical reaction took place between the base fluids and the metal oxide during the preparation. 

**Figure 9:**
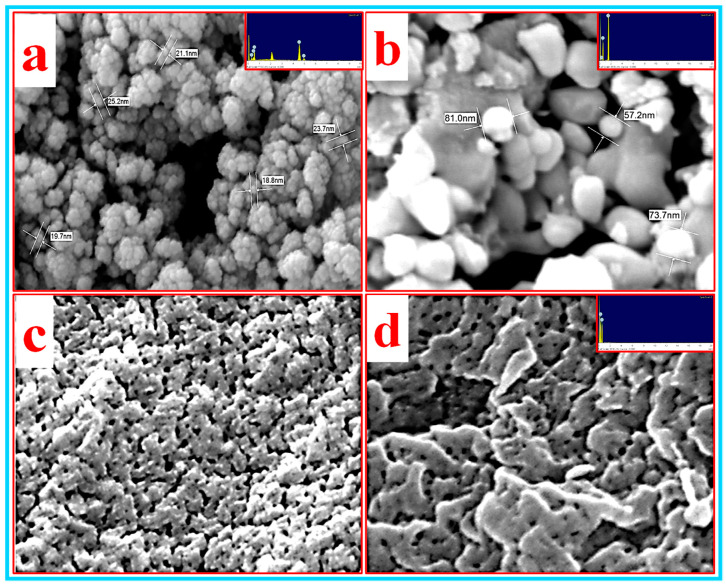
The FESEM micrograph of (**a**) TiO_2_ and (**b**) Al_2_O_3_ nanoparticles and the CNC (**c**) film and (**d**) powder at ×100,000 magnification with their respective EDX patterns (inset).

FESEM was used to investigate the surface morphological properties of all the samples and the results are shown in Figure 9a–d. From the FESEM image of the TiO_2_ nanoparticles (shown in Figure 9a), the shape of the individual particles is spherical with a diameter below 50 nm. These nanoparticles combined to form bigger particles which look like they are loosely bound or not properly agglomerated. Furthermore, the EDX analysis (inset) indicates the presence of Ti and O atoms in the sample. Figure 9b and the inset represents the FESEM image and the corresponding EDX pattern of the Al_2_O_3_ nanoparticles, respectively. The FESEM image depicts that primary particles are almost spherical in shape. These nanoparticles interconnected to each other form large particles (microparticles) that have irregular shapes. The small and bigger particles have diameters in the range of 50–90 nm and 1–5 µm, respectively. Furthermore, the elemental analysis of these particles confirms the presence of Al and O in the nanoparticles (inset). On the other hand, CNC was in the gel form which makes it difficult to analyze the morphological properties using FESEM. Therefore, two samples (i.e., film and powder) of CNC were prepared by drying for the FESEM analysis and the obtained results are shown in the inset of Figure 9c,d. It can be observed from both figures that no individual nanoparticles could be seen in both samples. However, the particles interconnected with each other formed a porous morphology which looks like a net. Nonetheless, the EDX analysis (inset of Figure 9d) confirmed the presence of the C and O atoms in the CNC nanoparticles.

### 4.2. Thermo-Physical Properties Evaluation

It was observed from the literature that the thermal conductivity of the nanofluids significantly increased on increasing the volume concentration of the suspended nanoparticles in the base fluid. For instance, the thermal conductivity enhancement was observed by Fani, Kalteh [58] with an increasing volume concentration of the nanoparticles. They reported that the collision between the particles intensified causing an increment in the Brownian diffusivity assisting which results in thermal conductivity enhancement. The thermal conductivity of TiO_2_, Al_2_O_3_, CNC, Al_2_O_3_/TiO_2_ and Al_2_O_3_/CNC nanofluids with different volume concentrations of 0.1%, 0.5% and 0.9% were measured and the results are shown in Figure 8a. It can be observed from the figure that the thermal conductivity of both the mono and hybrid nanofluids increases by increasing the volume concentration. It was found that the mono nanofluid (Al_2_O_3_) shows higher thermal conductivity improvement than the CNC and TiO_2_ nanofluids, due to the better thermal properties of Al_2_O_3_. Furthermore, it was shown that the Al_2_O_3_/CNC hybrid nanofluid exhibited a superior thermal conductivity than any other hybrid as well as mono nanofluids. However, the increasing thermal conductivity of all the nanofluids (mono and hybrid) followed the augmentation of the adding of nanoparticles into the base fluid. Therefore, the 0.9% volume concentration of the Al_2_O_3_/CNC and Al_2_O_3_/TiO_2_ show a higher thermal conductivity than the 0.5% and 0.1% volume concentration. 

**Figure 10:**
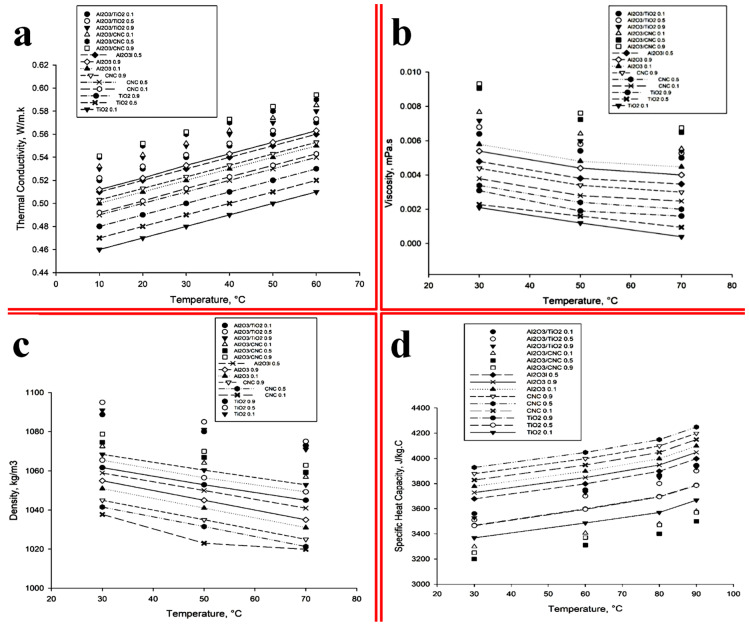
(**a**) Thermal conductivity of all the nanofluids vs. the temperature plot; (**b**) viscosity with respect to the temperature; (**c**) density comparison as a function of the temperature and the volume; and (**d**) comparison of the specific heat capacity of the mono and hybrid nanofluids with various volume concentrations.

In the present study, hybrid nanofluids exhibited better thermal conductivity than the mono nanofluids which may be due to the high kinetic energy generated by the high collisions of particles. Similar phenomenon were also observed by Esfe, Esfandeh [59] for ZnO/Multi-Walled Carbon NanoTube (MWCNT)/water–EG nanofluids where 28.1% higher thermal conductivity was obtained for hybrid nanofluid with 0.1% volume concentration than the single phase nanofluids at 50 °C. Huang, Wu [24] has also investigated the thermal conductivity enhancement of Al_2_O_3_ and MWCNTs dispersed into water-based hybrid nanofluid in a chevron plate heat exchanger and observed a better increment in the thermal conductivity than the Al_2_O_3_ nanofluid and water. Since the particles are capable of transferring heat directly from one to another at high temperature, therefore, high temperature increases the rate of heat transfer. At a high temperature, the Brownian motion of particles increases due to the high kinetic energy which then enhances the thermal conductivity. The maximum thermal conductivity was achieved at 60 °C in the present work. For instance, on increasing the temperature from 30 °C to 60 °C, the thermal conductivity of the Al_2_O_3_/CNC hybrid nanofluid increased from 0.57 to 0.59 W/m.K in a 0.9% volume fraction (Figure 10a). Similar work has also been reported in the literature. For example, Nabil, Azmi [60] observed an enhancement in the thermal conductivity of 22.8% for the TiO_2_-SiO_2_/water and EG hybrid nanofluid in a 3% volume fraction at 80 °C temperature which was much better than that observed by Hamid, Azmi [61] for the SiO_2_–TiO_2_/water and the EG hybrid nanofluid (22.1%) at 70 °C. Furthermore, Hamid, Azmi [61] has also reported that the thermal conductivity increased from 13.8% to 16% for the TiO_2_–SiO_2_/water and the EG hybrid nanofluid by a 1% volume fraction on increasing the temperature from 70 °C to 80 °C. A KD2 Pro Thermal Property Analyzer was used to evaluate the thermal conductivity followed the standard method entitled “American Society for Testing and Materials (ASTM) D7896-14 Standard Test Method for Thermal Conductivity, Thermal Diffusivity and Volumetric Heat Capacity of Engine Coolants and Related Fluids by Transient Hot Wire Liquid Thermal Conductivity Method”. 

The viscosity of all the nanofluids (mono and hybrid) were measured and the obtained results are shown in Figure 10b. It can be observed from the figure that the viscosity of the nanofluids is higher than the base fluid for both the mono and hybrid nanofluids. As the concentration increased, the viscosity also increased. The viscosity of the Al_2_O_3_ nanofluids at various volume fractions was found to be higher than the CNC and the TiO_2_ nanofluids. A similar effect of the volume concentration of the viscosity was also observed by Namburu, Kulkarni [62] and Fedele, Colla [34]. However, the viscosity of 0.1% volume concentration is higher than that of a 0.9% volume fraction of Al_2_O_3_ nanofluid, which does not support the previous literature on viscosity. Similarly, 0.1% CNC nanofluid exhibits a higher viscosity than a 0.5% CNC nanofluid as the packing of the particle caused movement restriction, where the addition of the CNC causes viscosity depreciation as per the trend observed for Al_2_O_3_ [63]. On the other hand, when more particles are added, the hybrid nanofluids such as Al_2_O_3_/CNC and Al_2_O_3_/TiO_2_ exhibit a higher viscosity than the mono nanofluids (Al_2_O_3,_ CNC and TiO_2_) with all the volume concentrations. However, the Al_2_O_3_/CNC nanofluid dominates over the viscosity of the Al_2_O_3_/TiO_2_ for all the volume concentrations. It can also be noticed from the figure that the viscosity decreases with increasing temperature. For instance, the viscosity of both hybrid (Al_2_O_3_/CNC and Al_2_O_3_/TiO_2_) nanofluids at all volume concentrations gradually decreased with the increasing temperature and found the lowest at a temperature of 70 °C, whereas the mono nanofluids (Al_2_O_3_, CNC, and TiO_2_) at all volume concentrations, except 0.1% and 0.5% TiO_2,_ exhibited the lowest viscosity at a temperature of 50 °C which showed an increasing trend at 70 °C. The effect of temperature on the viscosity of nanofluids was clarified by Li, Zou [14] based on the molecular viewpoint and reported that the intermolecular distance increases with a rising temperature which leads to the diminished pattern of the viscosity. The rotational viscometer was used to measure the viscosity that followed the standard method named “ASTM D2196-10 which is known as the standard test method for rheological properties of non-Newtonian materials by the rotational (Brookfield type) viscometer”.

The density of a nanofluid also plays an important role in the thermo-physical properties of nanofluids and depends on temperature [64]. The density of the mono and hybrid (TiO_2,_ Al_2_O_3,_ CNC, Al_2_O_3_/TiO_2_, and Al_2_O_3_/CNC) nanofluids was measured by varying the temperature as well as the volume concentration, and the obtained results are shown in Figure 10c. It can be noticed from the figure that the density of all the nanofluids (mono and hybrid) increased on adding nanoparticles into the base fluid and further gradually increased on loading the augmentation of the nanoparticles. However, only a 0.5% volume fraction of Al_2_O_3_ nanofluid exhibited a higher density than 0.9% Al_2_O_3_ nanofluid; this could have happened due to its size and unpredictable behavior [65]. Although the maximum density of all the mono and hybrid nanofluids was observed at a temperature of 30 °C, this gradually decreased until the temperature of 70 °C was reached. However, 0.1% CNC nanofluid contains a slightly higher density at a temperature of 70 °C than at 50 °C. Both hybrid nanofluids (i.e., Al_2_O_3_/TiO_2_ and Al_2_O_3_/CNC) showed the uppermost density value with regards to the mono nanofluids (Al_2_O_3,_ TiO_2,_ and CNC), although the Al_2_O_3_/TiO_2_ hybrid nanofluids portrayed a superior density than the other mono (Al_2_O_3,_ TiO_2,_ and CNC) and hybrid (Al_2_O_3_/CNC) nanofluids. The digital density meter was used to measure the density of the nanofluids and the hybrid nanofluids following the procedure of the “ASTM D4052-18 which is acknowledged as the standard test method for density, relative density and API gravity of liquids by digital density meter”.

Specific heat capacity is another vital thermo-physical property of nanofluids to observe their heat transfer performance. The specific heat capacity of the Al_2_O_3,_ CNC, TiO_2,_ Al_2_O_3_/CNC and the Al_2_O_3_/TiO_2_ nanofluids was measured as a function of temperature as well as a volume concentration and the obtained results are shown in Figure 10d. It was observed from the results that all the nanofluids (mono and hybrid) exhibited low and high specific heat at a temperature of 30 °C and 90 °C, respectively. Furthermore, the Al_2_O_3_/CNC hybrid nanofluid shows the lowest specific heat capacity than any other hybrid nanofluids. On the other hand, the CNC nanofluids (all volume concentrations) displayed the highest specific heat capacity compared to the other mono nanofluids. In the case of the CNC nanofluids, 0.5% CNC nanofluid exhibited the highest specific heat value compared to the rest of the CNC concentrations. On the contrary, the 0.1% Al_2_O_3_ nanofluid depicts the uppermost specific heat capacity compared to the other concentrations of the Al_2_O_3_ nanofluids. It was observed from the above discussion that these results were not consistent with the typical research proposal. However, the better specific heat capacity value exhibited by the CNC nanoparticles was a surprising result. Furthermore, the nanofluid with CNC nanoparticles showed the highest specific heat capacity compared to the nanofluids with hybrid nanoparticles at a temperature of 30 °C. Overall, it was noticed from Figure 10d that the specific heat capacity was directly and inversely proportional to the temperature and volume concentration. Similar results have also been observed by Zhou and Ni [28]. Basically, the volume concentration has a bigger impact than the temperature on the specific heat capacity measurement [66]. Moreover, the specific heat capacity was more effective in the heat transfer application than the thermal conductivity [67]. Therefore, the nanofluid with enhanced specific heat capacity was required for an efficient thermal exchange application. Differential scanning calorimetry (DSC) equipment was used to measure the specific heat of the nanofluids and the hybrid nanofluids following the “ASTM E1269-11(2018) Standard Test Method for Determining Specific Heat Capacity by Differential Scanning Calorimetry” method. 

Based on the results above discussed, the statistical method was used to optimize the nanofluid to be used as a thermal transport fluid in the automotive cooling system. As per the measurement procedure, the inlet temperature was kept constant at 70 °C and the obtained values of the thermo-physical measurement with a different volume concentration at 70 °C are tabulated in Table 3.

The obtained thermo-physical measurement values were used to determine the response optimizer in the Minitab 17 software and the optimized volume concentration from the statistical analytical tool was found to be 0.4893% which can be rounded up to 0.5%. The individual desirability value (d) determines the optimized setting of the single response. The inverse parabolic graph proves that the thermo-physical property results are within the limits of the obtained optimized volume concentration. In other words, the value obtained from the analysis was 0.6112 which was in good agreement with the 0.5% concentration of the analysis. It was observed from the literature that the increment in the specific heat capacity was important with respect to the thermal conductivity enhancement for the automotive cooling application, reported by Tomar and Tripathi [68]. Therefore, the CNC and CNC + Al_2_O_3_ nanofluids with a 0.5% volume concentration were carefully chosen as the thermal transport fluids to be compared with convectional ethylene glycol–water mixture (EG–W).

After finalizing the optimum concentration of nanofluids, the heat transfer and flow behavior measurement of the conventional EG-W mixture, the CNC and the hybrid nanofluid (Al_2_O_3_/CNC) were carried out by using the fabricated radiator test rig. The convection heat transfer, the experimental heat transfer coefficient and the temperature distribution profile were measured in a radiator for heat transfer analysis and it is vital to compare these characteristics with thermal transport fluids. Furthermore, the Reynolds number, the Nusselt number and the friction factor were estimated using formulas for the flow behavior analysis, which was important to identify the characteristics of the CNC, the Al_2_O_3_/CNC and the EG-W. The heat transfer applicability of the nanofluids can be concluded by comparing their heat transfer performance and their flow behavior as follows in the next sections.

**(a) Experimental heat transfer coefficient**: the temperature distribution obtained from the experiments and the measured thermal conductivity were used to determine the heat transfer coefficient using the following Equation (8):

(8) $$h\left(exp\right)=\frac{\dot{m}\;C_{p}}{A_{s}}\frac{\left(T_{\mathrm{in}}-T_{\mathrm{out}}\right)}{\left(T_{b}-T_{s}\right)}$$

In this formula, *h* denotes the heat transfer coefficient, *C_p_* is the specific heat capacity, *A*_s_ denotes the exposed surface area, *T*_in_ is the input temperature, *T_out_* is the outlet temperature, *T*_s_ is the wall temperature (solid) and *T*_b_ is bulk fluid temperature (liquid).

**Figure 11:**
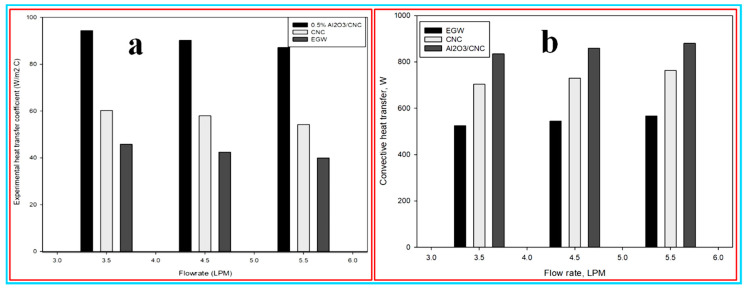
(**a**) Experimental heat transfer coefficient, and (**b**) Convective heat transfer as a function of the flow rate.

The obtained average experimental heat transfer coefficient as a function of the flow rate in (LPM unit) is shown in Figure 11a. It was observed from the figure that the experimental heat transfer coefficient for Al_2_O_3_/CNC, CNC and EG–W were found to be 94.93, 60.28 and 45.84 W/m^2^ °C at a 3.5 LPM flow rate and these values decrease up to 90.22, 57.98 and 42.5 W/m^2^ °C at a 4.5 LPM flow rate, respectively. The values of the experimental heat transfer coefficient for Al_2_O_3_/CNC, CNC and EG–W goes further down up to 87.23, 54.23 and 40.02 W/m^2^ °C at 5.5 LPM, respectively. It can be concluded from the obtained results that the experimental heat transfer coefficient directly depends upon the relation with the flow rate. Similar results have also been observed by Ali, Ali [69]. Namburu, Das [70] also investigated the heat transfer performance in a radiator test rig for an EG–W mixture with dispersed copper oxide (CuO) and reported that the heat transfer coefficient boosted up to 1.35 times more than the base fluid at a 20,000 Reynolds number. Moreover, the fan produced a drastic increment in the heat transfer coefficient value compared to the without a fan circumstance. The abnormal behavior in the high-transfer coefficient value of the Al_2_O_3_/CNC nanofluid can be better correlated with the high specific heat capacity and the thermal conductivity of the Al_2_O_3_/CNC rather than the CNC and EG-W. Generally, the rate of heat transfer affects the heat removal application. Therefore, the observed high relative heat transfer coefficient value indicates that the better heat removal can be obtained in Al_2_O_3_/CNC rather than CNC and EG-W at a low volumetric flow rate. Besides, the heat transfer coefficient value with the influence of a fan has a higher value than in circumstances without a fan. Indeed, the air velocity used during the measurement accelerates the rate of the heat removal in the radiator test rig.

**(b) Convection heat transfer**: the obtained convective heat transfer values for 0.5% Al_2_O_3_/CNC, 0.5% CNC and EG-W as a function of the flow rate is shown in Figure 11b. From the figure, the maximum convection heat transfer was found to be 880.42, 763.29 and 566.32 W for Al_2_O_3_/CNC, CNC and EG–W at 5.5 LPM, respectively. In other words, the 55.46% enhancement in the convective heat transfer was observed for Al_2_O_3_/CNC rather than for EG-W and 15.35% than the CNC at a 5.5 LPM flow rate. Furthermore, the convective heat transfers of 858.85 W for Al_2_O_3_/CNC, 729.94 W for CNC and 545.78 W for EG-W were measured at a flow rate of 4.5 LPM. The minimum value of the convective heat transfer, i.e., 835.38, 704.32 and 525.02 W for Al_2_O_3_/CNC, CNC and EG-W were measured at a 3.5 LPM flow rate. Based on the discussed results it can be concluded that the Al_2_O_3_/CNC exhibits a higher convective heat transfer, i.e., 15% more than the CNC and 50% more than the EG-W at all three flow rates. The high thermal conductivity and specific heat capacity of Al_2_O_3_/CNC were considered the main reasons for the high convective heat transfer in Al_2_O_3_/CNC.

**Figure 12:**
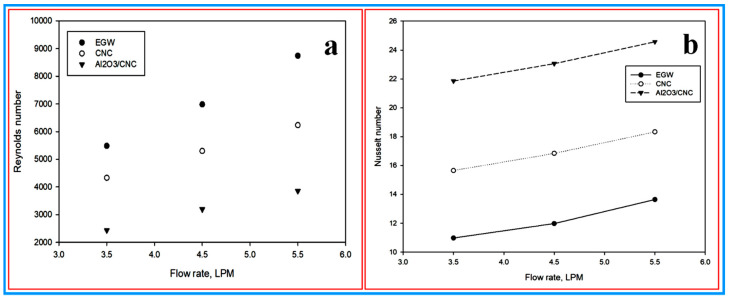
(**a**) Reynolds number vs. the flow rate and (**b**) the Nusselt number vs. the flow rate.

**(c) Reynolds number:** the Reynolds number is an important factor and it needs to be calculated to identify the type of flow regime in the radiator test rig. The Reynolds number was calculated using Equation (9) and the calculated Reynolds number as a function of the plotted flow rate is shown in Figure 12a. The results revealed that the maximum/minimum Reynolds number was calculated for Al_2_O_3_/CNC, CNC and EG–W which are 3852.32/2433.42, 6234.54/4329.43 and 8741.12/5483.83 at 5.5/3.5 LPM, respectively. From the results, it can be seen that the Reynolds number for all the above nanofluids have a proportional relation with the flow rate i.e., the Reynolds number increased when the flow rate was rising. Therefore, it can be concluded that the flow regime achieved by the Al_2_O_3_/CNC, CNC and EG–W can be considered as turbulent and remains similar inside the radiator at a varying flow rate between 3.5 and 5.5 LPM. The almost identical trend of the Reynolds number was also observed by Ali, Ali [69] for a ZnO nanofluid. Furthermore, Heris, Esfahany [9] has also investigated the properties of an Al_2_O_3_ nanofluid and obtained a lower value for the Reynolds number than for the base fluid. Basically, a low Reynolds number is more likely to correlate with the impact of a high viscous force than the inertial force in the nanofluid [71]. Therefore, the low value of the Reynolds number of the CNC may be due to the high dynamic viscosity value rather than the EG–W. Furthermore, the high density in the CNC results of the high inertial effect on the nanofluid plays an important role in determining the Reynolds number. In view of this, the Reynolds number increases with the flow rate which can be explained by the proportional relation of the Reynolds number to velocity:

(9) $$Re=\frac{\rho \;D\;\nu }{\mu }$$

where *v* is the flow velocity, *ρ* is the density, *µ* is the dynamic viscosity and *D* is the hydraulic diameter. These variables were measured using the instrumentation provided in the setup description (Section 3.1) and the thermo-physical properties evaluation (Section 4.2). 

**(d) Nusselt number**: the Nusselt number is also one of the vital parameters of the flow behavior of nanofluids. Basically, it is the ratio of the convective to conductive heat transfer across a boundary. In the present work, the Nusselt number was calculated for all the aforementioned nanofluids using Equation (10) and the results are shown in Figure 12b in the form of the Nusselt number vs. the flow rate plot. In the present work, the maximum obtained Nusselt numbers for the Al_2_O_3_/CNC, CNC and EG–W were 24.57, 18.34 and 13.64 at 5.5 LPM, whereas the minimum values were 21.86, 15.66 and 10.98 at a 3.5 LPM flow rate, respectively. From the graph, the Nusselt number has a proportional relation with the flow rate. The high influence of the convective heat transfer over the conductive heat transfer and the high experimental heat transfer coefficient value can be considered the reason for the higher value of the Nusselt number observed for the CNC than for the EG-W [26]. Therefore, it can be concluded that the value of the heat transfer coefficient was directly proportional to the Nusselt number:

(10) $$N_{u}=\frac{hD}{k}$$

where *h* is the heat transfer coefficient, *D* is the hydraulic diameter and *k* is the measured thermal conductivity of the different nanofluids. 

**Figure 13:**
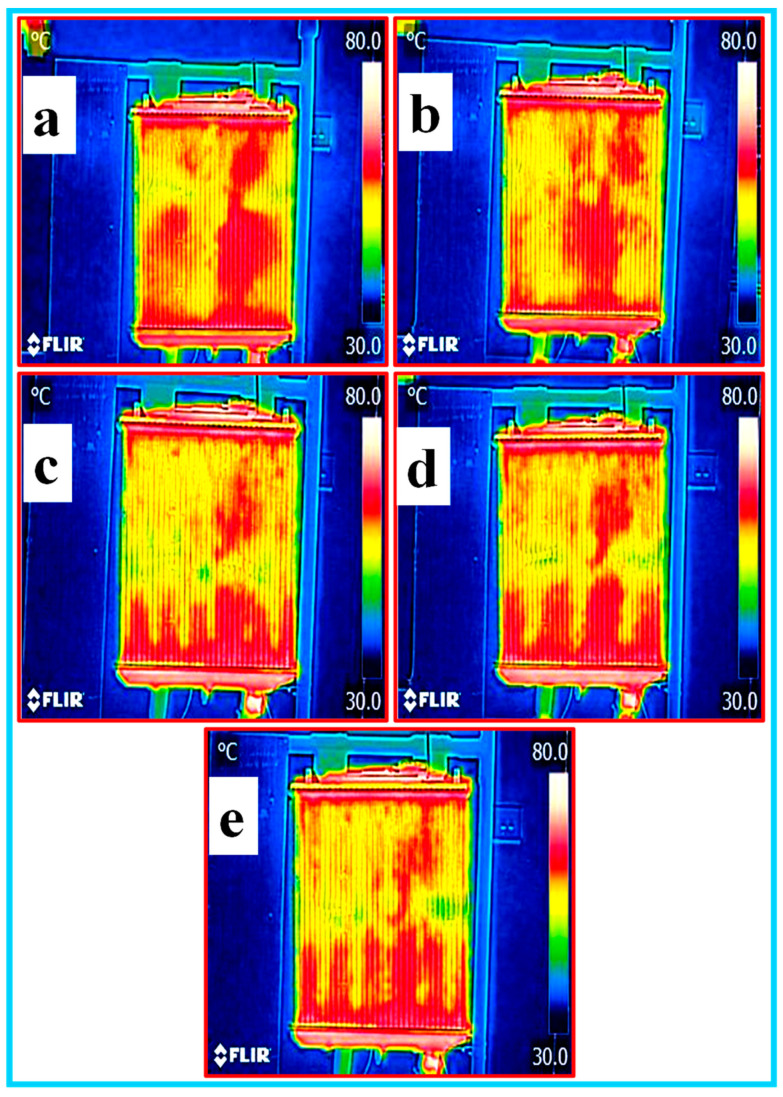
Thermal imaging of the base fluids (ethylene glycol–water mixture (EG–W)) in the radiator (**a**–**e**).

**Figure 14:**
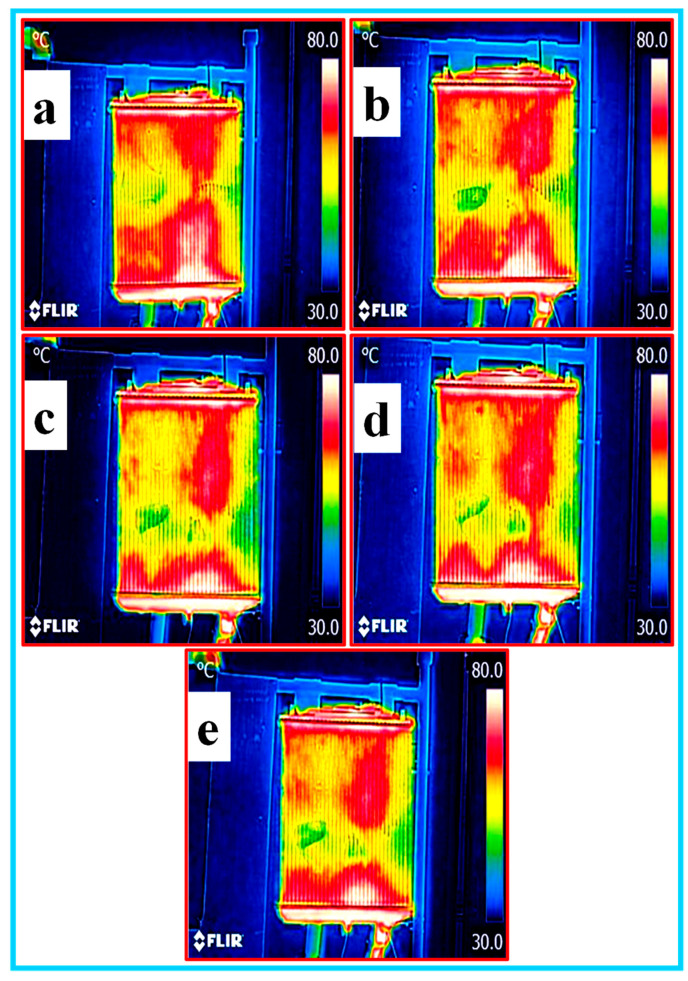
Thermal imaging of the Al_2_O_3_/CNC with a 0.5% volume concentration in the radiator (**a**–**e**).

**Figure 15:**
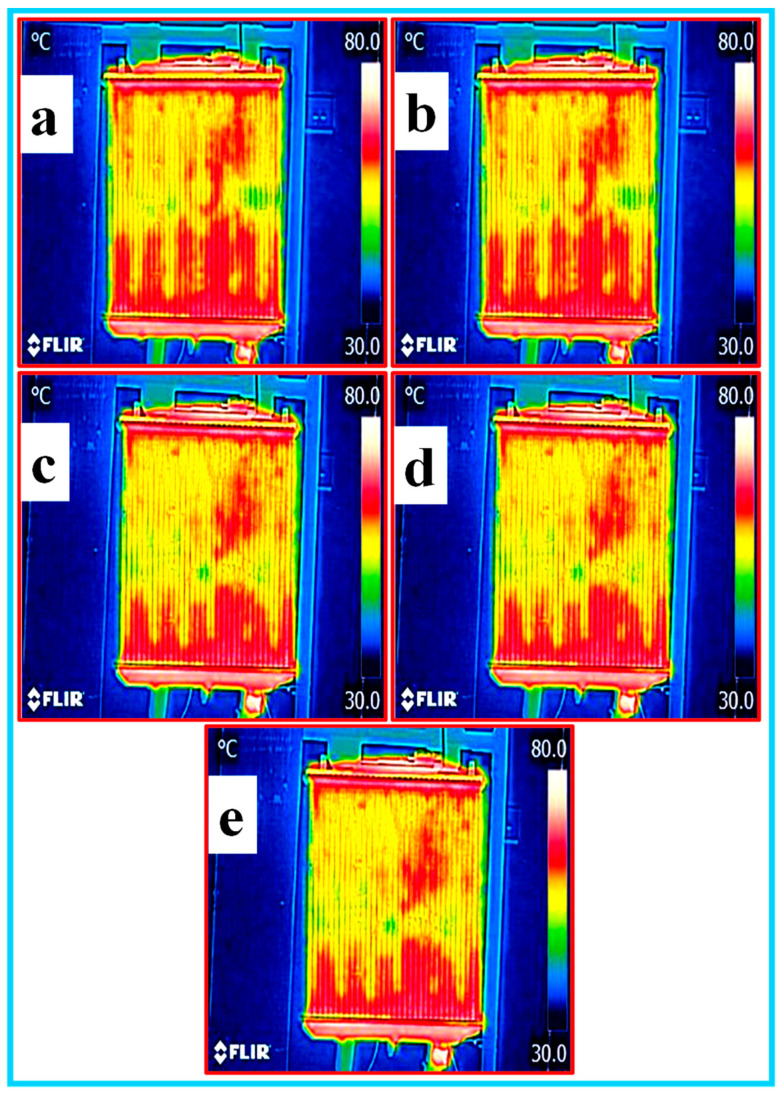
Thermal imaging of the CNC with a 0.5% volume concentration in the radiator (**a**–**e**).

**(e) Thermal heat analysis of nanofluids**: the thermal heat analysis of all the nanofluids was carried by capturing the images of the heat distribution of the fluid inside the radiator using thermal infrared camera FLIR model. The inside temperature of the radiator test rig was in the range of 30–70 °C during the image capturing. The thermal images of EG–W, Al_2_O_3_/CNC and CNC with a 0.5% volume concentration circulating in the radiator are shown in Figure 13a–e, Figure 14a–e and Figure 15a–e, respectively. The yellowish or green color in the radiator images reveals the absorption of heats during the measurement in the test rig. In view of this, it can be observed from the figure that the nanofluid covers the maximum area having a low temperature (green color), however, the highest heat dissipation occurred in the middle of the radiator. Among all the prepared nanofluids, it was observed that the Al_2_O_3_/CNC nanofluid absorbed the most heat. 

**Figure 16:**
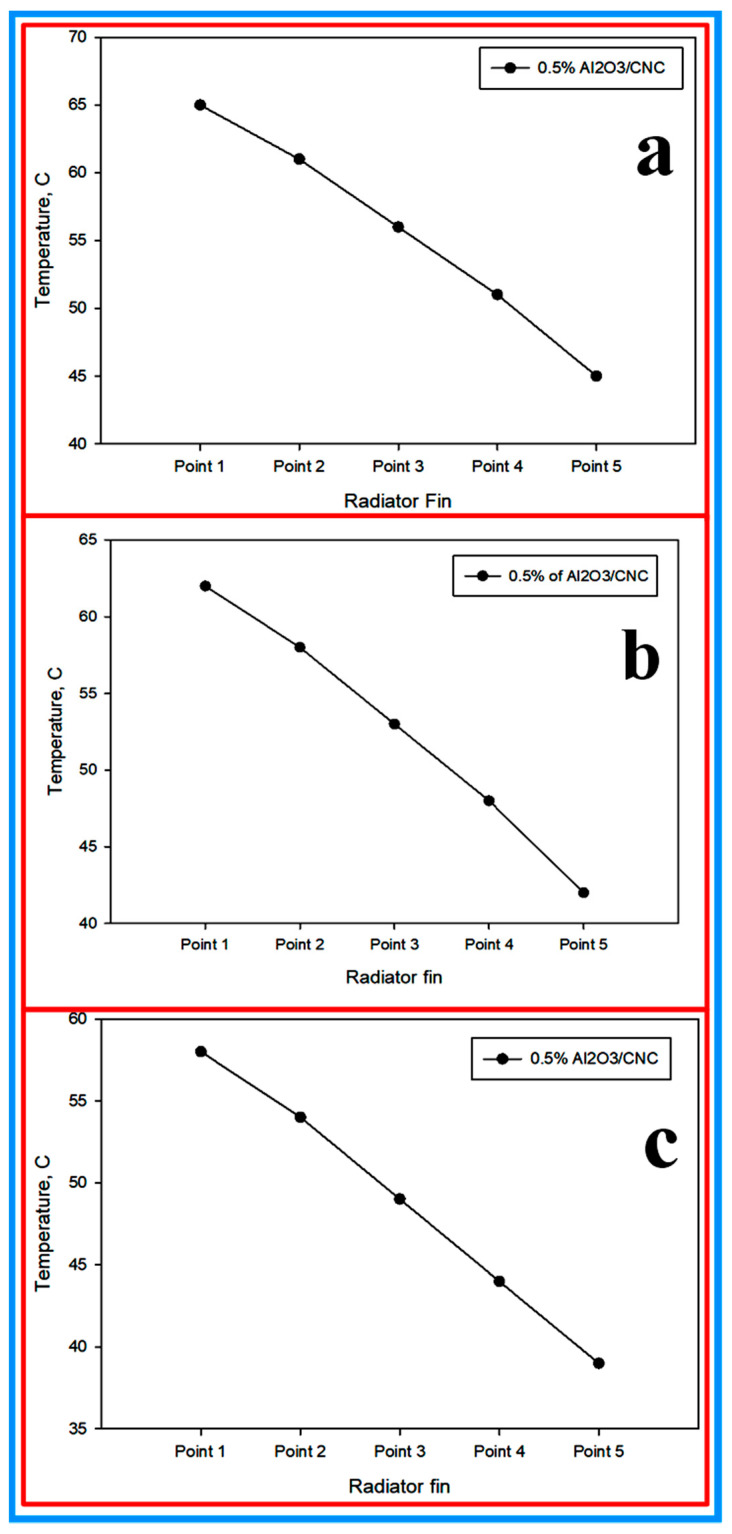
Temperature profile at the radiator fin at various flow rates, (**a**) 3.5 LPM, (**b**) 4.5 LPM, and (**c**) 5.5 LPM.

**(d) Temperature at radiator fin:** as per the above discussion, 0.5% volume concentration Al_2_O_3_/CNC was found to be better than the CNC and EG–W in terms of thermal heat properties, therefore, it was selected for further analysis. For the measurement, five points were chosen at the radiator fin to analyze the temperature at three different flow rates. This temperature was selected to get the average temperature on the fins. The temperature values at the 3.5, 4.5 and 5.5 LPM flow rates are shown in Figure 16a–c, respectively. It was observed that point 5 has less temperature compared to point 1. This was due to the heat transfer process occurring in the fins where the temperature is reduced.

## 5. Statistical Analysis

The response surface methodology (RSM) is a methodology of constructing approximations of the system behavior using results of the response analyses and calculating at a series of points in the variable space. The optimization of the RSM can be solved in the following three stages i.e., design of the experiment, building the model and the solution of the minimization problem according to the selected criterion. The concept of the response surface contained a dependent variable (y) which was also known as the response variable and several other independent variables *x*1, *x*2, …, *x*k. If all these variables are assumed to be measurable, the response surface (y) can be expressed as shown in Equation (11):

*y* = f (*x*1; *x*2; …; *x*k) (11)

For optimizing the response variable y, negligible error was assumed with the independent variables which were continuous and controllable during the measurement. On the contrary, a response or dependent variable was assumed to be a random variable. In the present work, low order polynomial such as first order and second order were employed in some regions of the independent variable.

**Figure 17:**
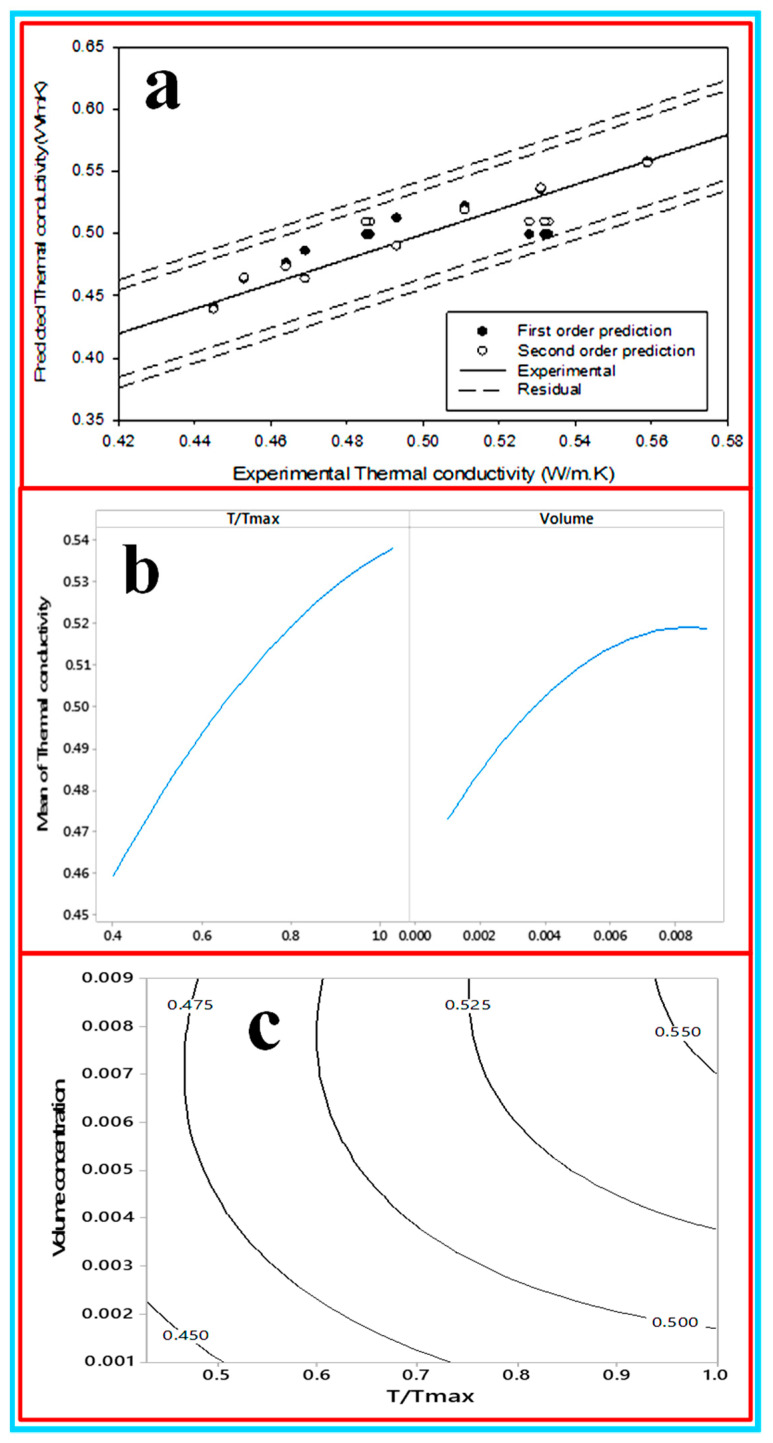
(**a**) Predicted thermal conductivity, (**b**) the factorial plot and (**c**) the contour plot for the thermal conductivity.

**(a) Development of first and second order of thermal conductivity of CNC/Al_2_O_3_ using RSM**: the effect of the considered factors on the compressive behavior of the samples was investigated by performing an analysis of variance (ANOVA) and the results are tabulated in Table 4. The objective of the ANOVA table is to investigate the importance of the parameters on the experiments. The model generated can be saved for future experimental process. The model can also predict the values of the response without running extra experiments. Basically, the ANOVA table was generated from the Minitab software. However, the main fundamental condition for obtaining it was the statistical equations that generated the values. The investigating factors are the temperature and the volume concentration, whereas the output factor was the thermal conductivity. The obtained *p*-value revealed the impact of the term. The level of the significant set to 0.05 permits choosing the parameters whose impact is not insignificant from a measurable perspective [72]. It shows that the temperature and volume concentration significantly affect the thermal conductivity. The temperature shows the dominant effect on the thermal conductivity since it contributed 50.46% and followed by volume 20.30% as shown in the ANOVA Table 4. These findings support that the particles are capable of transferring heat directly from one to another at a high temperature: the higher the kinetic energy at a high temperature, the more the rate of heat transfer increases. Figure 17a shows the predicted thermal conductivity with a 2–4% error. It shows that the quadratic equation predicts more closely compared with the linear equation. The contour plot and the factorial plot show in Figure 17b,c that the thermal conductivity increase with the increase in temperature and volume. The first-order and quadratic equation to predict the thermal conductivity are shown in Equations (12) and (13):

k_nf_ = 0.3825 + 0.1260 (T/T_max_) + 3.81ø (12)

k_nf_ = 0.3477 + 0.23(T/T_max_) +7.5ø − 0.108(T/T_max2_) − 833ø2 + 9.19(T/T_max_*ø) (13)

**Figure 18:**
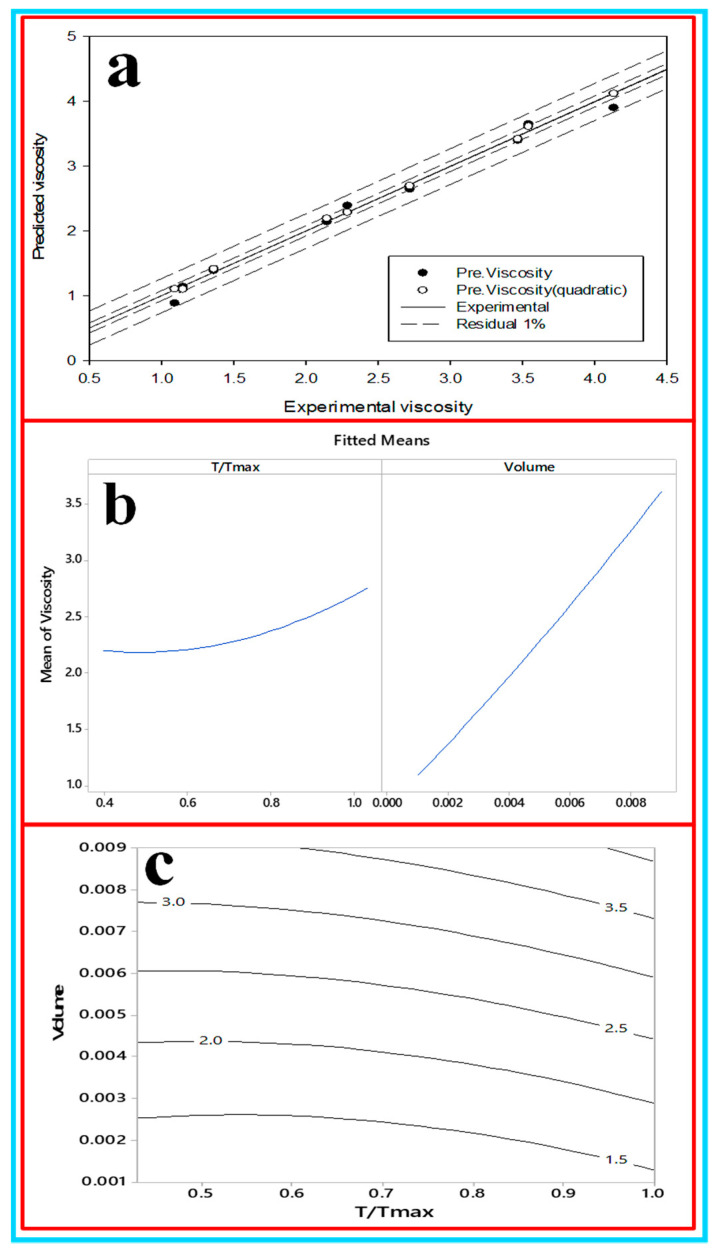
(**a**) Predicted viscosity, (**b**) the factorial plot and (**c**) the contour plot for the viscosity.

**(b) Development of the first and second order of viscosity of CNC/Al_2_O_3_ using RSM**: in order to investigate the influence of the considered factors on viscosity, the analysis of variance (ANOVA) was performed as shown in Table 5 where the investigated factors are the temperature and volume and the analyzed output factor was the viscosity. It shows that the temperature and volume have a significant effect on viscosity. Since a 95% confidence level was selected, the volume was the main dominant since it contributed 94.47% as shown in Table 5. This viscosity increasing phenomenon can be interpreted as the increasing nanoparticle concentration dispersed in the base fluid that improved the internal shear stress, subsequently increasing the viscosity [73]. The free volume in the nanofluid structure will increase and the internal friction forces between the molecules decrease [74]. The main effect plot shows that the high concentration enhances the viscosity of the liquid as shown in Figure 18a,b, and shows the predicted viscosity where the error was in the range of 2–10% and Figure 18c shows the decrement in the viscosity of the liquid. The first-order equation and quadratic to predict viscosity are shown in Equations (14) and (15):

Viscosity = 0.189 + 0.880 T/T_max_ + 314.1ø (14)

Viscosity = 1.478 − 2.287 T/T_max_ + 209.5ø+1.915 T/T_max_*T/T_max_ + 4302ø2+86.2 T/T_max_*ø (15)

**Figure 19:**
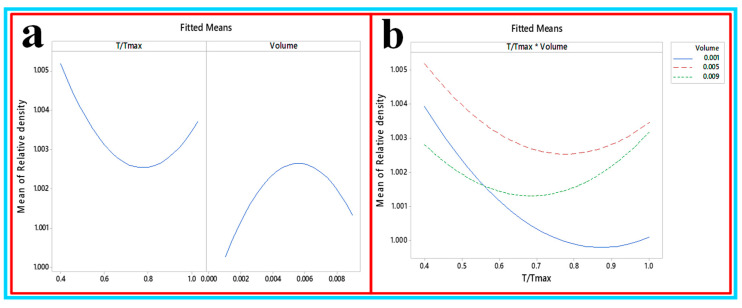
(**a**) Factorial plot and (**b**) interaction plot for the density.

**(c) Development of the first and second order of density of CNC/Al_2_O_3_ using RSM**: in order to investigate the influence of the considered factors on the density behavior of the samples, the analysis of variance (ANOVA) was performed as shown in Table 6. The investigated factors were the temperature and the concentration and the analyzed output factor was the density. It showed that none of the factors significantly affected the density. Figure 19a,b shows the factorial plot and the interaction plot. There was an interaction between 0.1 and 0.9 volume at 0.55 T/T_max_. The first-order and quadratic equation to predict the density are shown in Equations (16) and (17):

Relative density = 1.00349 − 0.00235 T/T_max_ + 0.134 ø (16)

Relative density = 1.01343 − 0.0334 T/T_max_ + 0.645 ø + 0.01868 T/T_max_2 − 113.4 ø2 + 0.872 T/T_max_* ø (17)

**Figure 20:**
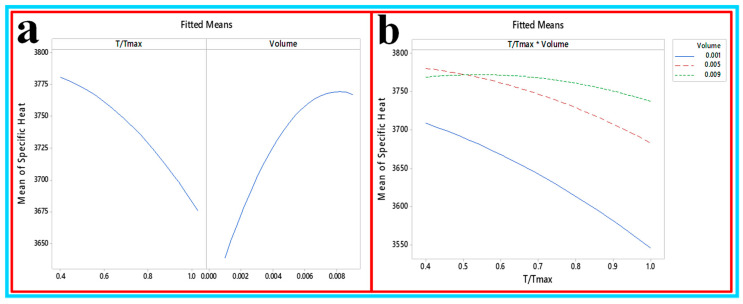
(**a**) Factorial plot and (**b**) interaction plot for specific heat.

**(d) Development of the first and second order of the specific heat of CNC/Al_2_O_3_ using RSM**: to investigate the influence of the considered factors on the roughness behavior of samples, the analysis of variance (ANOVA) was performed as shown in Table 7. It shows that none of the factors significantly affected the specific heat. The specific heat was mainly affected by the properties and phase of a given substance. It seems that other parameters such as the temperature and volume concentration did not influence the specific heat. Figure 20a,b shows the factorial plot and the interaction plot. There is an interaction between 0.5 and 0.9 volume at 0.5 T/T_max_. The first-order equation to predict the specific heat is shown in Equations (18) and (19):

Specific Heat = 3758 − 167 T/T_max_ + 16,072 ø (18)

Specific Heat = 3731 − 65 T/T_max_ + 22589ø -167 T/T_max_2 − 2,599,291 ø2 + 27,265 T/T_max_*ø (19)

## 6. Conclusions

The performance of the mono or hybrid metal oxide such as Al_2_O_3_ and TiO_2_ with or without plant base-extracted nanocellulose (CNC) with varying concentration as a better heat transfer nanofluid in comparison to distilled water as a radiator coolant was investigated comprehensively using experimental and numerical approaches. The CNC was dispersed in the base fluid of ethylene glycol (EG) and water (W) with a 60:40 ratio. The following conclusions can be drawn from the present study:

- The highest absorption peak was noticed in a 0.9% volume concentration of the TiO_2_, Al_2_O_3_, CNC, Al_2_O_3_/TiO_2_, and Al_2_O_3_/CNC nanofluids which indicated the better stability of the nanofluid suspension. The peak absorbance of the 0.9% volume fraction was much higher than the 0.5% volume concentration for the Al_2_O_3_, CNC and TiO_2_ nanofluids, while the discrepancy was less in the hybrid nanofluids between these concentrations.
- The hybrid nanofluids exhibited better thermal conductivity than the mono nanofluids. For instance, a better thermal conductivity improvement was shown by the mono Al_2_O_3_ nanofluids than the CNC and TiO_2_ nanofluids. On the other hand, a superior thermal conductivity was observed for Al_2_O_3_/CNC hybrid nanofluids as compared to other mono and hybrid (Al_2_O_3_/TiO_2_) nanofluids. The thermal conductivity of nanofluids (mono and hybrid) significantly increased with the increasing volume concentration of the nanoparticles suspended in the base fluid. Therefore, the higher thermal conductivity was observed for the Al_2_O_3_/CNC and Al_2_O_3_/TiO_2_ hybrid nanofluids with 0.9% volume concentration. Furthermore, the Al_2_O_3_/CNC hybrid nanofluid with 0.9% volume concentration showed a superior thermal conductivity compared to the other mono and hybrid nanofluids with all the volume concentrations.
- A higher viscosity was observed for the nanofluids than for the base fluid, which also increased with the increasing concentration of nanoparticles. Al_2_O_3_/CNC and Al_2_O_3_/TiO_2_ hybrid nanofluids exhibited higher viscosity than the mono nanofluids (Al_2_O_3_, CNC and TiO_2_) in all the volume concentrations. However, the Al_2_O_3_/CNC hybrid nanofluid dominated overt the other mono and hybrid nanofluids in terms of viscosity at all volume concentrations.
- It was observed that the density of all the nanofluids (Al_2_O_3_, TiO_2_, CNC, Al_2_O_3_/TiO_2_, and Al_2_O_3_/CNC) was increased when nanoparticles were added into the base fluid which further gradually increased with the loading augmentation of nanoparticles. However, only 0.5% volume fraction of the Al_2_O_3_ nanofluid exhibited a higher density. Although the maximum density of all the mono and hybrid nanofluids was observed at a temperature of 30 °C, which gradually decreased to a temperature of 70 °C. The Al_2_O_3_/TiO_2_ hybrid nanofluids portrayed a superior density than the other mono (Al_2_O_3_, TiO_2_ and CNC) and hybrid (Al_2_O_3_/CNC) nanofluids.
- The specific heat capacity of the mono and hybrid nanofluids was investigated by varying the temperature and volume concentration. A maximum and minimum specific heat were observed at temperatures of 30 °C and 90 °C for all the nanofluids, respectively. The CNC nanofluids (all volume concentrations) exhibited the highest specific heat capacity compared to the other mono nanofluids. Additionally, the lowest specific heat value was observed for the 0.9% CNC among all the concentrations. In both hybrid nanofluids, the Al_2_O_3_/CNC showed the lowest specific heat capacity. The optimized volume concentration from the statistical analytical tool was found to be 0.4893% which was rounded up to 0.5%.
- The nanofluid volume concentration with 0.5% (CNC/Al_2_O_3_ and CNC) was selected as the thermal transport fluid to be compared with the convectional EG-water mixture (EG-W). The experimental heat transfer coefficients for the Al_2_O_3_/CNC, CNC and EG-W were found to be 94.93, 60.28 W/m^2^ °C and 45.84 W/m^2^ °C at a 3.5 LPM flow rate and these values decreased up to 87.23 W/m^2^ °C, 54.23 W/m^2^ °C and 40.02 W/m^2^ °C at 5.5 LPM, respectively, which indicated that the experimental heat transfer coefficient directly depends upon the flow rate (LPM).

## Figures and Tables

**Table 1:** Parameters and description of the variables of the experiment.

Parameter	Description
Constant	Thermocouples points
	Heating and data-retrieving time period
	Radiator fan speed
	Position of 1 kW immerse heater
	Total volume of experimented nanofluids
Manipulated	Flow rates of nanofluids (3.5 L/min, 4.5 L/min and 5.5 L/min)

**Table 2:** Qualitative stability evaluation of the mono and hybrid nanofluids.

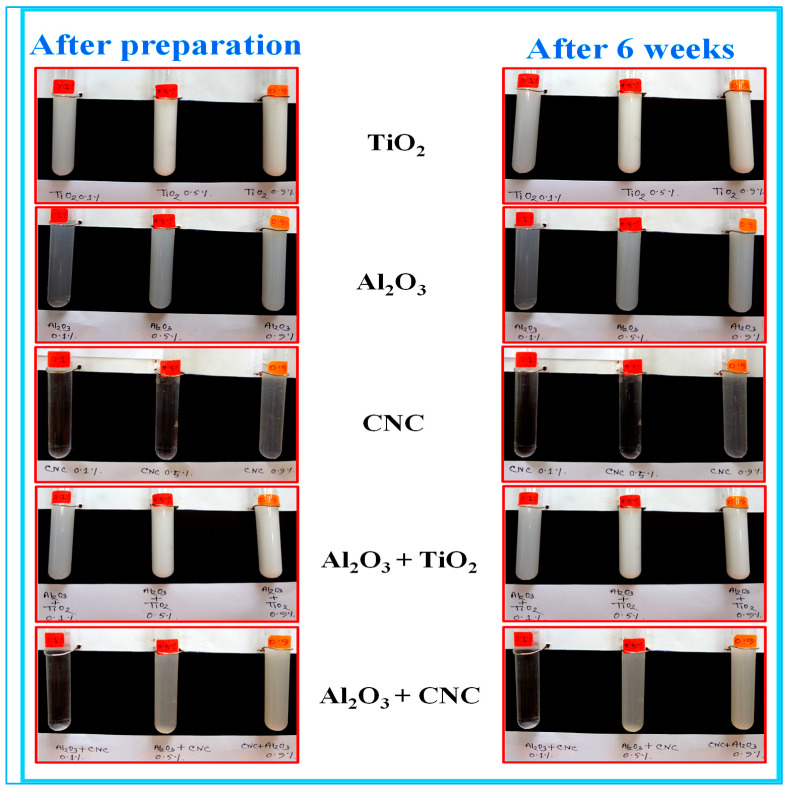

**Table 3:** Thermo-physical measurement of the varying volume concentration at 70 °C.

Volume Concentration (%)	Thermal Conductivity (W/m K)	Viscosity (mPa.s)	Density (kg/m^3^)	Specific Heat (J/kg °C)
0.1%	0.54	1.34	1049.34	3758.29
0.5%	0.55	2.06	1061.64	3636.26
0.9%	0.56	2.90	1084.14	3522.95

**Table 4:** ANOVA analysis for the thermal conductivity.

Items	Degree of Freedom	Contribution of the Parameters	F-Statistic	Probability-Value
Model	5	80.85%	5.91	0.019
Linear	2	70.75%	12.93	0.004
T/Tmax	1	50.46%	18.44	0.004
Volume	1	20.30%	7.42	0.03
Square	2	7.24%	1.32	0.326
T/Tmax*T/Tmax	1	4.05%	0.51	0.498
Volume*Volume	1	3.18%	1.16	0.316
2-Way Interaction	1	2.86%	1.05	0.34
T/Tmax*Volume	1	2.86%	1.05	0.34
Error	7	19.15%		
Lack-of-Fit	3	2.94%	0.24	0.864

**Table 5:** ANOVA table for viscosity.

Items	Degree of Freedom	Contribution of the Parameters	F-Statistic	Probability-Value
Model	5	99.85%	921.44	0
Linear	2	98.26%	2266.89	0
T/Tmax	1	3.78%	174.59	0
Volume	1	94.47%	4359.19	0
Square	2	1.20%	27.78	0
T/Tmax*T/Tmax	1	1.07%	31.06	0.001
Volume*Volume	1	0.13%	6.02	0.044
2-Way Interaction	1	0.39%	17.87	0.004
T/Tmax*Volume	1	0.39%	17.87	0.004
Error	7	0.15%		
Lack-of-Fit	3	0.15%		
Pure Error	4	0.00%		
Total	12	100.00%		

**Table 6:** ANOVA table for density.

Items	Degree of Freedom	Contribution of the Parameters	F-Statistic	Probability-Value
Model	5	67.95%	2.97	0.094
Linear	2	15.26%	1.67	0.256
T/Tmax	1	9.32%	2.04	0.197
Volume	1	5.93%	1.3	0.292
Square	2	39.02%	4.26	0.062
T/Tmax*T/Tmax	1	7.73%	4.83	0.064
Volume*Volume	1	31.29%	6.84	0.035
2-Way Interaction	1	13.67%	2.99	0.128
T/Tmax*Volume	1	13.67%	2.99	0.128
Error	7	32.05%		
Lack-of-Fit	3	23.59%	3.72	0.118
Pure Error	4	8.46%		
Total	12	100.00%		

**Table 7:** ANOVA for specific heat.

Items	Degree of Freedom	Contribution of the Parameters	F-Statistic	Probability-Value
Model	5	24.47%	0.45	0.799
Linear	2	18.85%	0.87	0.459
T/Tmax	1	6.69%	0.62	0.457
Volume	1	12.16%	1.13	0.324
Square	2	3.72%	0.17	0.845
T/Tmax*T/Tmax	1	1.37%	0.02	0.883
Volume*Volume	1	2.34%	0.22	0.655
2-Way Interaction	1	1.90%	0.18	0.687
T/Tmax*Volume	1	1.90%	0.18	0.687
Error	7	75.53%		
Lack-of-Fit	3	20.52%	0.5	0.704
Pure Error	4	55.01%		
Total	12	100.00%		

## Nomenclature

ρ	Density (kg/m^3^)
ϕ	Volumetric concentration of particles (%)
$C_{p}$	Specific heat (J/kg °C)
k	Thermal conductivity (W/m-K)
w	Mass fraction
V	Volume concentration
μ	Viscosity (mPa.s)
D	Linear dimension (m)
ν	Velocity (m/s)
h	Convection heat transfer coefficient (W/m^2^ °C)
L	Length (m)
$R_{e}$	Reynolds number
$N_{u}$	Nusselt number
Subscripts	
*f*	Fluid
*nf*	Nanofluid
*p*	Particle
